# Opportunities and challenges in long-read sequencing data analysis

**DOI:** 10.1186/s13059-020-1935-5

**Published:** 2020-02-07

**Authors:** Shanika L. Amarasinghe, Shian Su, Xueyi Dong, Luke Zappia, Matthew E. Ritchie, Quentin Gouil

**Affiliations:** 1 Epigenetics and Development Division, The Walter and Eliza Hall Institute of Medical Research, Parkville, 3052 Australia; 2 Department of Medical Biology, The University of Melbourne, Parkville, 3010 Australia; 3 Bioinformatics, Murdoch Children’s Research Institute, Parkville, 3052 Australia; 4 School of Biosciences, Faculty of Science, The University of Melbourne, Parkville, 3010 Australia; 5 School of Mathematics and StatisticsThe University of Melbourne, Parkville, 3010 Australia

**Keywords:** Long-read sequencing, Data analysis, PacBio, Oxford Nanopore

## Abstract

Long-read technologies are overcoming early limitations in accuracy and throughput, broadening their application domains in genomics. Dedicated analysis tools that take into account the characteristics of long-read data are thus required, but the fast pace of development of such tools can be overwhelming. To assist in the design and analysis of long-read sequencing projects, we review the current landscape of available tools and present an online interactive database, long-read-tools.org, to facilitate their browsing. We further focus on the principles of error correction, base modification detection, and long-read transcriptomics analysis and highlight the challenges that remain.

## Introduction

Long-read sequencing, or third-generation sequencing, offers a number of advantages over short-read sequencing [1, 2]. While short-read sequencers such as Illumina’s NovaSeq, HiSeq, NextSeq, and MiSeq instruments [3–5]; BGI’s MGISEQ and BGISEQ models [6]; or Thermo Fisher’s Ion Torrent sequencers [7, 8] produce reads of up to 600 bases, long-read sequencing technologies routinely generate reads in excess of 10 kb [1].

Short-read sequencing is cost-effective, accurate, and supported by a wide range of analysis tools and pipelines [9]. However, natural nucleic acid polymers span eight orders of magnitude in length, and sequencing them in short amplified fragments complicates the task of reconstructing and counting the original molecules. Long reads can thus improve de novo assembly, mapping certainty, transcript isoform identification, and detection of structural variants. Furthermore, long-read sequencing of native molecules, both DNA and RNA, eliminates amplification bias while preserving base modifications [10]. These capabilities, together with continuing progress in accuracy, throughput, and cost reduction, have begun to make long-read sequencing an option for a broad range of applications in genomics for model and non-model organisms [2, 11].

Two technologies currently dominate the long-read sequencing space: Pacific Biosciences’ (PacBio) single-molecule real-time (SMRT) sequencing and Oxford Nanopore Technologies’ (ONT) nanopore sequencing. We henceforth refer to these simply as SMRT and nanopore sequencing. SMRT and nanopore sequencing technologies were commercially released in 2011 and 2014, respectively, and since then have become suitable for an increasing number of applications. The data that these platforms produce differ qualitatively from second-generation sequencing, thus necessitating tailored analysis tools.

Given the broadening interest in long-read sequencing and the fast-paced development of applications and tools, the current review aims to provide a description of the guiding principles of long-read data analysis, a survey of the available tools for different tasks as well as a discussion of the areas in long-read analysis that require improvements. We also introduce the complementary open-source catalogue of long-read analysis tools: long-read-tools.org. The long-read-tools.org database allows users to search and filter tools based on various parameters such as technology or application.

## The state of long-read sequencing and data analysis

Nanopore and SMRT long-read sequencing technologies rely on very distinct principles. Nanopore sequencers (MinION, GridION, and PromethION) measure the ionic current fluctuations when single-stranded nucleic acids pass through biological nanopores [12, 13]. Different nucleotides confer different resistances to the stretch of nucleic acid within the pore; therefore, the sequence of bases can be inferred from the specific patterns of current variation. SMRT sequencers (RSII, Sequel, and Sequel II) detect fluorescence events that correspond to the addition of one specific nucleotide by a polymerase tethered to the bottom of a tiny well [14, 15].

Read length in SMRT sequencing is limited by the longevity of the polymerase. A faster polymerase for the Sequel sequencer introduced with chemistry v3 in 2018 increased the read lengths to an average 30-kb polymerase read length. The library insert sizes amenable to SMRT sequencing range from 250 bp to 50 kbp. Nanopore sequencing provides the longest read lengths, from 500 bp to the current record of 2.3 Mb [16], with 10–30-kb genomic libraries being common. Read length in nanopore sequencing is mostly limited by the ability to deliver very high-molecular weight DNA to the pore and the negative impact this has on run yield [17]. Basecalling accuracy of reads produced by both these technologies have dramatically increased in the recent past, and the raw base-called error rate is claimed to have been reduced to < 1% for SMRT sequencers [18] and < 5% for nanopore sequences [17].

While nanopore and SMRT are true long-read sequencing technologies and the focus of this review, there are also synthetic long-read sequencing approaches. These include linked reads, proximity ligation strategies, and optical mapping [19–28], which can be employed in synergy with true long reads.

**Fig. 1:**
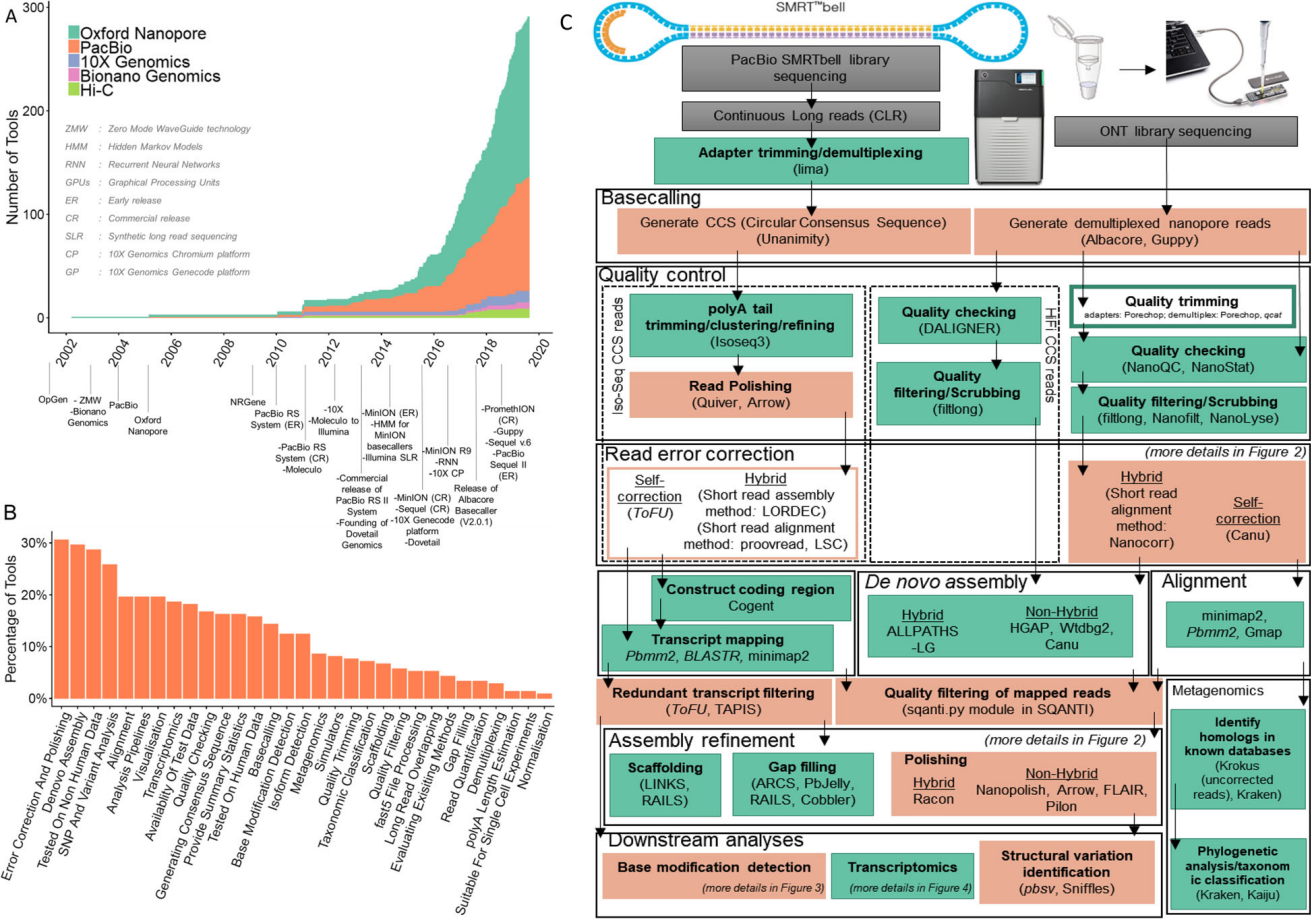
Overview of long-read analysis tools and pipelines. **a** Release of tools identified from various sources and milestones of long-read sequencing. **b** Functional categories. **c** Typical long-read analysis pipelines for SMRT and nanopore data. Six main stages are identified through the presented workflow (i.e. basecalling, quality control, read error correction, assembly/alignment, assembly refinement, and downstream analyses). The green-coloured boxes represent processes common to both short-read and long-read analyses. The orange-coloured boxes represent the processes unique to long-read analyses. Unfilled boxes represent optional steps. Commonly used tools for each step in long-read analysis are within brackets. Italics signify tools developed by either PacBio or ONT companies, and non-italics signify tools developed by external parties. Arrows represent the direction of the workflow

With the potential for accurately assembling and re-assembling genomes [17, 29–32], methylomes [33, 34], variants [18], isoforms [35, 36], haplotypes [37–39], or species [40, 41], tools to analyse the sequencing data provided by long-read sequencing platforms are being actively developed, especially since 2011 (Fig. 1a).

A search through publications, preprints, online repositories, and social media identified 354 long-read analysis tools. The majority of these tools are developed for nanopore read analyses (262) while there are 170 tools developed to analyse SMRT data (Fig. 1a). We categorised them into 31 groups based on their functionality (Fig. 1b). This identified trends in the evolution of research interests: likely due to the modest initial throughput of long-read sequencing technologies, the majority of tools were tested on non-human data; tools for de novo assembly, error correction, and polishing categories have received the most attention, while transcriptome analysis is still in early stages of development (Fig. 1b).

We present an overview of the analysis pipelines for nanopore and SMRT data and highlight popular tools (Fig. 1c). We do not attempt to provide a comprehensive review of tool performance for all long-read applications; dedicated benchmark studies are irreplaceable, and we refer our readers to those when possible. Instead, we present the principles and potential pitfalls of long-read data analysis with a focus on some of the main types of downstream analyses: structural variant calling, error correction, detection of base modifications, and transcriptomics.

## Basecalling

The first step in any long-read analysis is basecalling, or the conversion from raw data to nucleic acid sequences (Fig. 1c). This step receives greater attention for long reads than short reads where it is more standardised and usually performed using proprietary software. Nanopore basecalling is itself more complex than SMRT basecalling, and more options are available: of the 26 tools related to basecalling that we identified, 23 relate to nanopore sequencing.

During SMRT sequencing, successions of fluorescence flashes are recorded as a movie. Because the template is circular, the polymerase may go over both strands of the DNA fragment multiple times. SMRT basecalling starts with segmenting the fluorescence trace into pulses and converting the pulses into bases, resulting in a continuous long read (also called polymerase read). This read is then split into subreads, where each subread corresponds to 1 pass over the library insert, without the linker sequences. Subreads are stored as an unaligned BAM file. From aligning these subreads together, an accurate consensus circular sequence (CCS) for the insert is derived [42]. SMRT basecallers are chiefly developed internally and require training specific to the chemistry version used. The current basecalling workflow is ccs [43].

Nanopore raw data are current intensity values measured at 4 kHz saved in fast5 format, built on HDF5. Basecalling of nanopore reads is an area of active research, where algorithms are quickly evolving (neural networks have supplanted HMMs, and various neural networks structures are being tested [44]) as are the chemistries for which they are trained. ONT makes available a production basecaller (Guppy, currently) as well as development versions (Flappie, Scrappie, Taiyaki, Runnie, and Bonito) [45]. Generally, the production basecaller provides the best accuracy and most stable performance and is suitable for most users [46]. Development basecallers can be used to test features, for example, homopolymer accuracy, variant detection, or base modification detection, but they are not necessarily optimised for speed or overall accuracy. In time, improvements make their way into the production basecaller. For example, Scrappie currently maps homopolymers explicitly [47].

Independent basecaller with different network structures are also available, most prominently Chiron [48]. These have been reviewed and their performance evaluated elsewhere [13, 46, 49]. The ability to train one’s own basecalling model opens the possibility to improve basecalling performance by tailoring the model to the sample’s characteristics [46]. As a corollary, users have to keep in mind that the effective accuracy of the basecaller on their data set may be lower than the advertised accuracy. For example, ONT’s basecallers are currently trained on a mixture of human, yeast, and bacterial DNA; their performance on plant DNA where non-CG methylation is abundant may be lower [50]. As the very regular updates to the production Guppy basecaller testify, basecalling remains an active area of development.

## Errors, correction, and polishing

Both SMRT and nanopore technologies provide lower per read accuracy than short-read sequencing. In the case of SMRT, the circular consensus sequence quality is heavily dependent on the number of times the fragment is read—the depth of sequencing of the individual SMRTbell molecule (Fig. 1c)—a function of the length of the original fragment and longevity of the polymerase. With the Sequel v2 chemistry introduced in 2017, fragments longer than 10 kbp were typically only read once and had a single-pass accuracy of 85–87% [51]. The late 2018 v3 chemistry increases the longevity of the polymerase (from 20 to 30 kb for long fragments). An estimated four passes are required to provide a CCS with Q20 (99% accuracy) and nine passes for Q30 (99.9% accuracy) [18]. If the errors were non-random, increasing the sequencing depth would not be sufficient to remove them. However, the randomness of sequencing errors in subreads, consisting of more indels than mismatches [52–54], suggests that consensus approaches can be used so that the final outputs (e.g. CCS, assembly, variant calls) should be free of systematic biases. Still, CCS reads retain errors and exhibit a bias for indels in homopolymers [18].

On the other hand, the quality of nanopore reads is independent of the length of the DNA fragment. Read quality depends on achieving optimal translocation speed (the rate of ratcheting base by base) of the nucleic acid through the pore, which typically decreases in the late stages of sequencing runs, negatively affecting the quality [55]. Contrary to SMRT sequencing, a nanopore sequencing library is made of linear fragments that are read only once. In the most common, 1D sequencing protocol, each strand of the dsDNA fragment is read independently, and this single-pass accuracy is the final accuracy for the fragment. By contrast, the 1D^2^ protocol is designed to sequence the complementary strand in immediate succession of up to 75% of fragments, which allows the calculation of a more accurate consensus sequence for the library insert. To date, the median single-pass accuracy of 1D sequencing across a run can reach 95% (manufacturer’s numbers [56]). Release 6 of the human genomic DNA NA12878 reference data set reports 91% median accuracy [17]. 1D^2^ sequencing can achieve a median consensus accuracy of 98% [56]. An accurate consensus can also be derived from linear fragments if the same sequence is present multiple times: the concept of circularisation followed by rolling circle amplification for generating nanopore libraries is similar to SMRT sequencing, and subreads can be used to determine a high-quality consensus [57–59]. ONT is developing a similar linear consensus sequencing strategy based on isothermal polymerisation rather than circularisation [56].

Indels and substitutions are frequent in nanopore data, partly randomly but not uniformly distributed. Low-complexity stretches are difficult to resolve with the current (R9) pores and basecallers [56], as are homopolymer sequences. Measured current is a function of the particular *k*-mer residing in the pore, and because translocation of homopolymers does not change the sequence of nucleotides within the pore, it results in a constant signal that makes determining homopolymer length difficult. A new generation of pores (R10) was designed to increase the accuracy over homopolymers [56]. Certain *k*-mers may differ in how distinct a signal they produce, which can also be a source of systematic bias. Sequence quality is of course intimately linked to the basecaller used and the data that has been used to train it. Read accuracy can be improved by training the basecaller on data that is similar to the sample of interest [46]. ONT regularly release chemistry and software updates that improve read quality: 4 pore versions were introduced in the last 3 years (R9.4, R9.4.1, R9.5.1, R10.0), and in 2019 alone, there were 12 Guppy releases. PacBio similarly updates hardware, chemistry, and software: the last 3 years have seen the release of 1 instrument (Sequel II), 4 chemistries (Sequel v2 and v3; Sequel II v1 and v2), and 4 versions of the SMRT-LINK analysis suite.

**Fig. 2:**
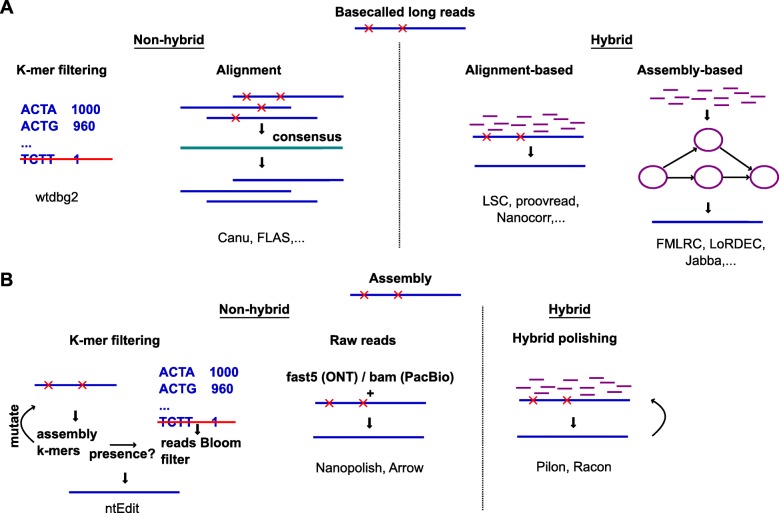
Paradigms of error correction (**a**) and polishing (**b**). Errors in long reads and assembly are denoted by red crosses. Non-hybrid methods only require long reads, while hybrid methods additionally require accurate short reads (purple)

Although current long-read accuracy is generally sufficient to uniquely determine the genomic origin of the read, certain applications require high base-level accuracy, including de novo assembly, variant calling, or defining intron-exon boundaries [54]. Two groups of methods to error correct long-reads can be employed: methods that only use long reads (non-hybrid) and methods that leverage the accuracy of additional short-read data (hybrid) (Fig. 2). Zhang et al. recently reviewed and benchmarked 15 of these long-read error correction methods [60], while Fu et al. focused on 10 hybrid error correction tools [61]. Lima et al. benchmarked 11 error correction tools specifically for nanopore cDNA reads [62].

In non-hybrid methods, all reads are first aligned to each other and a consensus is used to correct individual reads (Fig. 2a). These corrected reads can then be taken forward for assembly or other applications. Alternatively, because genomes only contain a small subset of all possible *k*-mers, rare *k*-mers in a noisy long-read data set are likely to represent sequencing errors. Filtering out these rare *k*-mers, as the wtdbg2 assembler does [63], effectively prevents errors from being introduced in the assembly (Fig. 2a).

Hybrid error correction methods can be further classified according to how the short reads are used. In alignment-based methods, the short reads are directly aligned to the long reads, to generate corrected long reads (Fig. 2a). In assembly-based methods, the short reads are first used to build a de Bruijn graph or assembly. Long reads are then corrected by aligning to the assembly or by traversing the de Bruijn graph (Fig. 2a). Assembly-based methods tend to outperform alignment-based methods in correction quality and speed, and FMLRC [64] was found to perform best in the two benchmark studies [60, 61].

After assembly, the process of removing remaining errors from contigs (rather than raw reads) is called ‘polishing’. One strategy is to use SMRT subreads through Arrow [65] or nanopore current traces through Nanopolish [66], to improve the accuracy of the consensus (Fig. 2b). For nanopore data, polishing while also taking into account the base modifications (as implemented for instance in Nanopolish [66]) further improves the accuracy of an assembly [46]. Alternatively, polishing can be done with the help of short reads using Pilon [67], Racon [68], or others, often in multiple rounds [50, 69, 70] (Fig. 2b). The rationale for iterative hybrid polishing is that as errors are corrected, previously ambiguously mapped short reads can be mapped more accurately. While certain pipelines repeat polishing until convergence (or oscillatory behaviour, where the same positions are changed back and forth between each round), too many iterations can decrease the quality of the assembly, as measured by the BUSCO score [71]. To increase scalability, ntEdit foregoes alignment in favour of comparing the draft assembly’s *k*-mers to a thresholded Bloom filter built from the sequencing reads [72] (Fig. 2b).

Despite continuous improvements in the accuracy of long reads, error correction remains indispensable in many applications. We identified 62 tools that are able to carry out error correction. There is no silver bullet, and correcting an assembly requires patience and careful work, often combining multiple tools (e.g. Racon, Pilon, and Nanopolish [50]). Adding to the difficulty of the absence of an authoritative error correction pipeline, certain tools do not scale well for deep sequencing or large genomes [50]. Furthermore, most tools are designed with haploid assemblies in mind. Allelic variation, repeats, or gene families may not be correctly handled.

## Detecting structural variation

While short reads perform well for the identification of single nucleotide variants (SNVs) and small insertion and deletions (indels), they are not well suited to the detection of larger sequence changes [73]. Collectively referred to as structural variants (SVs), insertions, deletions, duplications, inversions, or translocations that affect ≥ 50 bp [74] are more amenable to long-read sequencing [75, 76] (Fig 1c). Because of these past technical limitations, structural variants have historically been under-studied despite being an important source of diversity between genomes and relevant for human health [77, 78].

The ability of long reads to span repeated elements or repetitive regions provides unique anchors that facilitate de novo assembly and SV calling [73]. Even relatively short (5 kb) SMRT reads can identify structural variants in the human genome that were previously missed by short-read technologies [79]. Obtaining deep coverage of mammalian-sized genomes with long reads remains costly; however, modest coverage may be sufficient: 8.6 × SMRT sequencing [14] and 15–17 × nanopore sequencing [80, 81] have been shown to be effective in detecting pathogenic variants in humans. Heterozygosity or mosaicism naturally increase the coverage requirements.

Evaluating the performance of long-read SV callers is complicated by the fact that benchmark data sets may be missing SVs in their annotation [73, 77], especially when it comes only from short reads. Therefore, validation of new variants has to be performed via other methods. Developing robust benchmarks is an ongoing effort [82], as is devising solutions to visualise complex, phased variants for critical assessment [82, 83].

For further details on structural variant calling from long-read data, we refer the reader to two recent reviews: Mahmoud et al. [73] and Ho et al. [77].

## Detecting base modifications

In addition to the canonical A, T, C, and G bases, DNA can contain modified bases that vary in nature and frequency across organisms and tissues. *N*-6-methyladenine (6mA), 4-methylcytosine (4mC), and 5-methylcytosine (5mC) are frequent in bacteria. 5mC is the most common base modification in eukaryotes, while its oxidised derivatives 5-hydroxymethylcytosine (5hmC), 5-formylcytosine (5fC), and 5-carboxycytosine (5caC) are detected in certain mammalian cell types but have yet to be deeply characterised [84–88]. Still, more base modifications that result from DNA damage occur at a low frequency [87].

The nucleotides that compose RNA are even more varied. Over 150 modified bases have been documented to date [89, 90]. These modifications also have functional roles, for example, in mRNA stability [91], transcriptional repression [92], and translational efficiency [93]. However, most RNA modifications remain ill-characterised due to technological limitations [94]. Aside from the modifications to standard bases, base analogues may also be introduced to nucleic acids, such as the thymidine analogue BrdU which is used to track genomic replication [95].

Mapping of nucleic acid modifications has traditionally relied on specific chemical treatment (e.g. bisulfite conversion that changes unmethylated cytosines to uracils [96]) or immunoprecipitation followed by sequencing [97]. The ability of the long-read platforms to sequence native nucleic acids provides the opportunity to determine the presence of many more modifications, at base resolution in single molecules, and without specialised chemistries that can be damaging to the DNA [98]. Long reads thus allow the phasing of base modifications along individual nucleic acids, as well as their phasing with genetic variants, opening up opportunities in exploring epigenetic heterogeneity [34, 99]. Long reads also enable the analysis of base modifications in repetitive regions of the genome (centromeres or transposons), where short reads cannot be mapped uniquely.

**Fig. 3:**
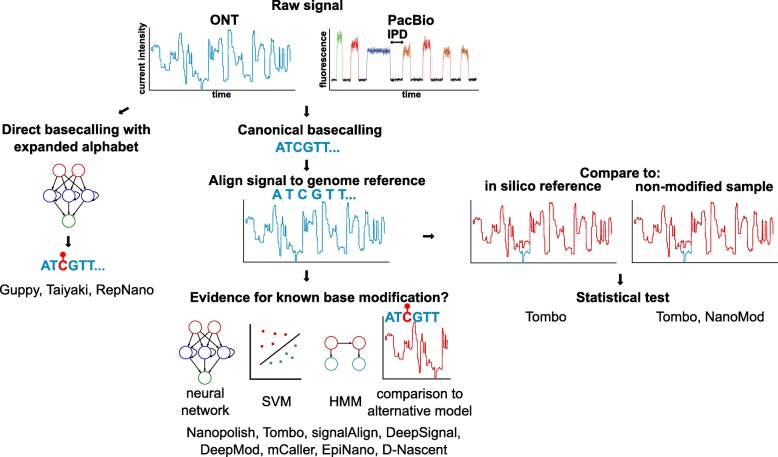
Methods to detect base modifications in long-read sequencing. Base modifications can be inferred from their effect on the current intensity (nanopore) and inter-pulse duration (IPD, SMRT). Strategies to call base modifications in nanopore sequencing and the corresponding tools are further depicted

In SMRT sequencing, base modifications in DNA or RNA [100, 101] are inferred from the delay between fluorescence pulses, referred to as interpulse duration (IPD) [98] (Fig. 3). Base modifications impact the speed at which the polymerase progresses, at the site of modification and/or downstream. Comparison with the signal from an in silico or non-modified reference (e.g. amplified DNA) suggests the presence of modified bases [102, 103]. It is notably possible to detect 6mA, 4mC, 5mC, and 5hmC DNA modifications, although at different sensitivity. Reliable calling of 6mA and 4mC requires 25 × coverage per strand, whereas 250 × is required for 5mC and 5hmC, which have subtler impacts on polymerase kinetics [102]. Such high coverage is not realistic for large genomes and does not allow single-molecule epigenetic analysis. Coverage requirements can be reduced by conjugating a glucose moeity to 5hmC, which gives a stronger IPD signal during SMRT sequencing [102, 103]. Polymerase dynamics and base modifications can be analysed directly via the SMRT Portal, or for more advanced analyses with R-kinetics, kineticsTools or basemods [104]. SMALR [99] is dedicated to the detection of base modifications in single SMRT reads.

In nanopore sequencing, modified RNA or DNA bases affect the flow of the current through the pore differently than non-modified bases, resulting in signal shifts (Fig. 3). These shifts can be identified post-basecalling and post-alignment with three distinct methods: (a) without prior knowledge about the modification (de novo) by comparing to an in silico reference [105], or a control, non-modified sample (typically amplified DNA) [105, 106]; (b) using a pre-trained model [66, 107, 108] (Fig. 3, Table 1); and (c) directly by a basecaller using an extended alphabet [45, 109].

**Table 1:** Tools and strategies to detect base modifications in Nanopore data (*HMM* hidden Markov model, *HPD* hierarchical Dirichlet process, *CNN* convolutional neural network, *LSTM* long short-term memory, *RNN* recurrent neural network, *SVM* support vector machine)

Tool	Base modifications	Strategy	Reference
Guppy	5mCpG, 5mC (Dcm), 6mA (Dam)	Basecall	[45]
Taiyaki	–	Basecall	[45]
RepNano	BrdU	Basecall	[109]
D-Nascent	BrdU	HMM	[95]
Nanopolish	5mCpG	HMM	[66]
Megalodon	6mA, 5mCpG	HMM	[45]
signalAlign	6mA, 5mC, 5hmC	HMM-HDP	[107]
DeepSignal	6mA (Dam), 5mCpG	Neural network (CNN + classifier)	[110]
DeepMod	6mA, 5mCpG	Neural network (LSTM-RNN)	[111]
mCaller	6mA, 5mCpG	Neural network classifier	[108]
Tombo	6mA (DNA), 5mC (RNA, DNA), de novo	Statistical test	[105]
NanoMod	de novo	Statistical test	[106]
EpiNano	m6A (RNA)	SVM	[112]

De novo approaches, as implemented by Tombo [105] or NanoMod [106], allow the discovery of modifications and modified motifs by statistically testing the deviation of the observed signal relative to a reference. However these methods suffer from a high false discovery rate and are not reliable at the single-molecule level. The comparison to a control sample rather than an in silico reference increases the accuracy of detection, but requires the sequencing of twice as many sample as well as high coverage to ensure that genomic segments are covered by both control and test sample reads. De novo calling of base modifications is limited to highlighting regions of the genomes that may contain modified bases, without being able to reveal the precise base or the nature of the modification.

Pre-trained models interrogate specific sites and classify the data as supporting a modified or unmodified base. Nanopolish [66] detects 5mC with a hidden Markov model, which in signalAlign [107] is combined with a hierarchical Dirichlet process, to determine the most likely *k*-mer (modified or unmodified). D-NAscent [95] utilises an approach similar to Nanopolish to detect BrdU incorporation, while EpiNano uses support vector machines (SVMs) to detect RNA m6A. Recent methods use neural network classifiers to detect 6mA and 5mC (mCaller [108], DeepSignal [110], DeepMod [111]). The accuracy of these methods is upwards of 80% but varies between modifications and motifs. Appropriate training data is crucial and currently a limiting factor. Models trained exclusively on samples with fully methylated or unmethylated CpGs will not perform optimally on biological samples with a mixture of CpG and mCpGs, or 5mC in other sequence contexts [66, 105]. Low specificity is particularly problematic for low abundance marks. m6A is present at 0.05% in mRNA [113, 114]; therefore, a method testing all adenosines in the transcriptome with sensitivity and specificity of 90% at the single-molecule, single-base level would result in an unacceptable false discovery rate of 98%.

Direct basecalling of modified bases is a recent addition to ONT’s basecaller Guppy, currently limited to 5mC in the CpG context. A development basecaller, Taiyaki [45], can be trained for specific organisms or base modifications. RepNano can basecall BrdU in addition to the four canonical DNA bases [109]. Two major bottlenecks in the creation of modification-ready basecallers are the need for appropriate training data and the combinatorial complexity of adding bases to the basecalling alphabet. There is also a lack of tools for the downstream analysis of base modifications: most tools output a probability that a certain base is modified, while traditional differential methylation algorithms expect binary counts of methylated and unmethylated bases.

## Analysing long-read transcriptomes

Alternative splicing is a major mechanism increasing the complexity of gene expression in eukaryotes [115, 116]. Practically, all multi-exon genes in humans are alternatively spliced [117, 118], with variations between tissues and between individuals [119]. However, fragmented short reads cannot fully assemble nor accurately quantify the expressed isoforms, especially at complex loci [120, 121]. Long-read sequencing provides a solution by ideally sequencing full-length transcripts. Recent studies that used bulk, single-cell, or targeted long-read sequencing suggest that our best transcript annotations are still missing vast numbers of relevant isoforms [122–126]. As noted above, sequencing native RNA further provides the opportunity to better characterise RNA modifications or other characteristics such as poly-A tail length. Despite its many promises, analysis of long-read transcriptomes remains challenging. Few of the existing tools for short-read RNA-seq analysis are able to appropriately deal with the high error rate of long reads, necessitating the development of dedicated tools and extensive benchmarks. Although recently, the field of long-read transcriptomics is rapidly expanding, we tallied 36 tools related to long-read transcriptome analysis (Fig. 1b).

**Fig. 4:**
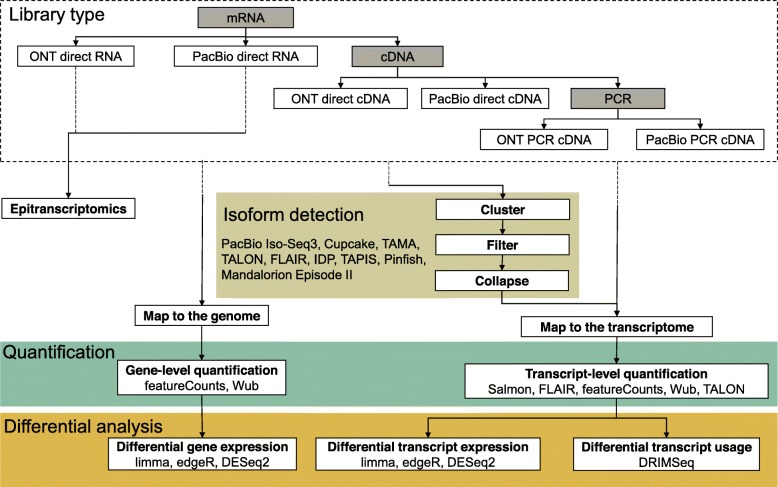
Types of transcriptomic analyses and their steps. The choice of sequencing protocol amongst the six available workflows affects the type, characteristics, and quantity of data generated. Only direct RNA sequencing allows epitranscriptomic studies, but SMRT direct RNA sequencing is a custom technique that is not fully supported. The remaining non-exclusive applications are isoform detection, quantification, and differential analysis. The dashed lines in arrows represent upstream processes to transcriptomics

Most long-read isoform detection tools work by clustering aligned and error-corrected reads into groups and collapsing these into isoforms, but the detailed implementations differ between tools (Fig. 4). PacBio’s Iso-Seq3 [127, 128] is the most mature pipeline for long-read transcriptome analysis, allowing the assembly of full-length transcripts. It performs pre-processing for SMRT reads, de novo discovery of isoforms by hierarchical clustering and iterative merging, and polishing. Cupcake [129] provides scripts for downstream analysis such as collapsing redundant isoforms and merging Iso-Seq runs from different batches, giving abundance information as well as performing junction analysis. In the absence of a reference genome, Iso-Seq can assemble a transcriptome, but transcripts from related genes may be merged [130] as a trade-off for correcting reads with a high error rate. Furthermore, the library preparation for Iso-Seq usually requires size fractionation, which makes absolute and relative quantification difficult. The per-read cost remains high, making well-replicated differential expression study designs prohibitively expensive.

Alternative isoform detection pipelines such as IsoCon [130], SQANTI [131], and TALON [132] attempt to mitigate the erroneous merging of similar transcripts of the Iso-Seq pipeline. IsoCon and SQANTI specifically work with SMRT data while TALON is a technology-independent approach. IsoCon uses the full-length transcripts from Iso-Seq to perform clustering and partial error correction and identify candidate transcripts without losing potential true variants within each cluster. SQANTI generates quality control reports for SMRT Iso-Seq data and detects and removes potential artefacts. TALON, on the other hand, relies heavily on the GENCODE annotation. Since both IsoCon and TALON focus on the human genome, they may not perform equally well with genomes from non-model organisms. A number of alternative isoform annotation pipelines for SMRT and/or nanopore data have recently emerged, such as FLAIR [133], Tama [134], IDP [122], TAPIS [135], Mandalorion Episode II [36, 57], and Pinfish [136]. Some of them use short reads to improve exon junction annotation. However, their accuracy has not yet been extensively tested.

In addition to high error rates, potential coverage biases are currently not explicitly taken into account by long-read transcriptomic tools. In ONT’s direct RNA sequencing protocol, transcripts are sequenced from the 3^′^ to the 5^′^ end; therefore, any fragmentation during the library prep, or pore blocking, results in truncated reads. In our experience, it is common to see a coverage bias towards the 3^′^ end of transcripts, which can affect isoform characterisation and quantification. Methods that sequence cDNA will also show these coverage biases due to fragmentation and pore-blocking (for nanopore data), compounded by non-processivity of the reverse transcriptase [124], more likely to stall when it encounters RNA modifications [137]. Finally, the length-dependent or sequence-dependent biases introduced by protocols that rely on PCR are currently not well characterised nor accounted for.

To quantify the abundance of transcripts or genes, several methods can be used (Fig. 4). Salmon’s [138] quasi-mapping mode quantifies reads directly against a reference index, and its alignment-based mode instead works with aligned sequences. The Wub package [139] also provides a script for read counting. The featureCounts [140] function from the Subread package [141, 142] supports long-read gene level counting. The FLAIR [133] pipeline provides wrappers for quantifying FLAIR isoform usage across samples using minimap2 or Salmon. Of course, for accurate transcript-level quantification, these methods rely on a complete and accurate isoform annotation; this is currently the difficult step.

Two types of differential analyses can be run: gene level or transcript level (Fig. 4). Transcript-level analyses may be further focused on differential transcript usage (DTU), where the gene may overall be expressed at the same level between two conditions, but the relative proportions of isoforms may vary. The popular tools for short-read differential gene expression analysis, such as limma [143], edgeR [144, 145], and DESeq2 [146], can also be used for long-read differential isoform or gene expression analyses. DRIMSeq [147] can perform differential isoform usage analysis using the Dirichlet-multinomial model. One difference between short- and long-read counts is that for the latter, counts per million (cpm) are effectively transcripts per million (tpm), whereas for short reads (and random fragmentation protocols), transcript length influences the number of reads, and therefore, cpms need scaling by transcript length to obtain tpms. The biological interpretation of differential isoform expression strongly depends on the classification of the isoforms, for example, whether the isoforms code for the same or different proteins or whether premature stop codons make them subject to nonsense-mediated decay. This is currently not well integrated into the analyses.

## Combining long reads, synthetic long reads, and short reads

Assemblies based solely on long reads generally produce highly complete and contiguous genomes [148–150]; however, there are many situations where short reads or reads generated from synthetic long-read technology further improve the results [151–153].

Different technologies can intervene at different scales: short reads ensure base-level accuracy, high-quality 5–15-kb SMRT reads generate good contigs, while ultra-long (100 kb+) nanopore reads, optical mapping or Hi-C improve scaffolding of the contigs into chromosomes [11, 17, 154–157]. Combining all of these technologies in a single genomic project would be costly. Instead, combinations of subsets are frequent, in particular, nanopore/SMRT with short-read sequencing [50, 152, 153, 158], although other combinations can be useful. Nanopore assembly of wild strains of *Drosophila melanogaster* supported by scaffolds generated from Hi-C corrected two misalignments of contigs in the reference assembly [154]. Optical maps helped resolve misassembly of SMRT-based chromosome level contigs of three plant relatives of *Arabidopsis thaliana*, where unrelated parts of the genome were erroneously linked [155].

For structural variation or base modification detection, obtaining orthogonal support from SMRT and nanopore data is valuable to confirm discoveries and limit false positives [77, 108, 159]. The error profiles of SMRT and nanopore sequencing are not identical—though both technologies experience difficulty around homopolymers—combining them can draw on their respective strengths.

Certain tools such as Unicycler [160] integrate long- and short-read data to produce hybrid assemblies, while other tools have been presented as pipelines to achieve this purpose (e.g. Canu, Pilon, and Racon in the ont-assembly-polish pipeline [45]). Still, combining tools and data types remains a challenge, usually requiring intensive manual integration.

## long-read-tools.org: a catalogue of long-read sequencing data analysis tools

The growing interest in the potential of long reads in various areas of biology is reflected by the exponential development of tools over the last decade (Fig. 1a). There are open-source static catalogues (e.g. github.com/B-UMMI/long-read-catalog), custom pipelines developed by individual labs for specific purposes (e.g. Search results from GitHub), and others that attempt to generalise them for a wider research community [46]. Being able to easily identify what tools exist—or do not exist—is crucial to plan and perform best-practice analyses, build comprehensive benchmarks, and guide the development of new software.

For this purpose, we introduce long-read-tools.org, a timely database that comprehensively collates tools used for long-read data analysis. Users can interactively search tools categorised by technology and intended type of analysis. In addition to true long-read sequencing technologies (SMRT and nanopore), we include synthetic long-read strategies (10X linked reads, Hi-C, and Bionano optical mapping). The fast-paced evolution of long-read sequencing technologies and tools also means that certain tools become obsolete. We include them in our database for completeness but indicate when they have been superseded or are no longer maintained.

long-read-tools.org is an open-source project under the MIT License, whose code is available through GitHub [161]. We encourage researchers to contribute new database entries of relevant tools and improvements to the database, either directly via the GitHub repository or through the submission form on the database webpage.

## Discussion

At the time of writing, for about USD1500, one can obtain around 30 Gbases of ≥ 99% accurate SMRT CCS (1 Sequel II 8M SMRT cell) or 50–150 Gbases of noisier but potentially longer nanopore reads (1 PromethION flow cell). While initially, long-read sequencing was perhaps most useful for assembly of small (bacterial) genomes, the recent increases in throughput and accuracy enable a broader range of applications. The actual biological polymers that carry genetic information can now be sequenced in their full length or at least in fragments of tens to hundreds of kilobases, giving us a more complete picture of genomes (e.g. telomere-to-telomere assemblies, structural variants, phased variations, epigenetics, metagenomics) and transcriptomes (e.g. isoform diversity and quantity, epitranscriptomics, polyadenylation).

These advances are underpinned by an expanding collection of tools that explicitly take into account the characteristics of long reads, in particular, their error rate, to efficiently and accurately perform tasks such as preprocessing, error correction, alignment, assembly, base modification detection, quantification, and species identification. We have collated these tools in the long-read-tools.org database.

The proliferation of long-read analysis tools revealed by our census makes a compelling case for complementary efforts in benchmarking. Essential to this process is the generation of publicly available benchmark data sets where the ground truth is known and whose characteristics are as close as possible to those of real biological data sets. Simulations, artificial nucleic acids such as synthetic transcripts or in vitro-methylated DNA, resequencing, and validation endeavours will all contribute to establishing a ground truth against which an array of tools can be benchmarked. In spite of the rapid iteration of technologies, chemistries, and data formats, these benchmarks will encourage the emergence of best practices.

A recurrent challenge in long-read data analysis is scalability. For instance in genome assembly, Canu [69] produces excellent assemblies for small genomes but takes too long to run for large genomes. Fast processing is crucial to enable parameter optimisation in applications that are not yet routine. The recently released wtdbg2 [63], TULIP [70], Shasta [162], Peregrine [163], Flye [164], and Ra [165] assemblers are orders of magnitude faster and are quickly being adopted. Similarly, for mapping long reads, minimap2’s speed, in addition to its accuracy, has contributed to its fast and wide adoption. Nanopolish [66] is popular both for assembly correction and base modification detection; however, it is slow on large data sets. The refactoring of its call-methylation function in f5c tool greatly facilitates work with large genomes or data sets [166].

Beyond data processing speed, scalability is also impacted by data generation, storage, and integration. Nanopore sequencing presents the fastest turnaround time. Once DNA is extracted, sequencing is underway in a matter of minutes to hours, and the PromethION sequencer provides adjustable high throughput with individually addressable parallel flow cells. All other library preparation procedures are more labour intensive, and sequencing may have to await pooling to fill a run, and flow cells need to be run in succession rather than in parallel. The raw nanopore data is however extremely voluminous (about 20 bytes per base), leading to substantial IT costs for large projects. SMRT movies are not saved for later re-basecalling, and the sequence and kinetic information takes up a smaller 3.5 bytes per base. Furthermore, hybrid methods incorporating strengths from other technologies such as optical mapping (Bionano, OpGen) and Hi-C add to the cost and analytical complexity of genomic projects. For these, manual data integration is a significant bottleneck, but the rewards are worth the effort.

Despite increasing accuracy of both SMRT and nanopore sequencing platforms, error correction remains an important step in long-read analysis pipelines. Published assemblies that omit careful error correction are likely to predict many spurious truncated proteins [167]. Hybrid error correction, leveraging the accuracy of short reads, is still outperforming long-read-only correction [60]. Modern short-read sequencing protocols require small input amounts (some even scale down to single cells) so sample amount is usually not a barrier to combining short- and long-read sequencing. Removing the need for short reads, and higher coverage via improvements in non-hybrid error correction tools and/or long-read sequencing accuracy, would reduce the cost, length, and complexity of genomic projects.

The much anticipated advances in epigenetics/epitranscriptomics promised by long-read sequencing are still in development. Many modifications, including 5mC, do not influence the SMRT polymerase’ dynamics sufficiently to be detected at a useful sensitivity (5mC requires 250 × coverage). In this case, software improvements are unlikely to yield significant gains, and improvements in sequencing chemistries are probably required [168]. Nanopore sequencing appears more amenable to the detection of a wide array of base modifications (to date: 5mCG, BrdU, 6mA), but the lack of ground truth data to train models and the combinatorial complexity of introducing multiple alternative bases are hindering progress towards a goal of seamless basecalling from an extended alphabet of canonical and non-canonical bases. Downstream analyses, in particular, differential methylation, exploiting the phasing of base modifications, as well as visualisation, suffer from a dearth of tools.

The field of long-read transcriptomics is equally in its infancy. To date, the Iso-Seq pipeline has been used to build catalogues of transcripts in a range of species [128, 169, 170]. Nanopore reads-based transcriptomes are more recent [10, 171–173], and work is still needed to understand the characteristics of these data (e.g. coverage bias, sequence biases, reproducibility). Certain isoform assembly pipelines predict a large number of unannotated isoforms requiring validation and classification. Even accounting for artefacts and transcriptional noise, these early studies reveal an unexpectedly large diversity in isoforms. Benchmark data and studies will be required in addition to atlas-type sequencing efforts to generate high-quality transcript annotations that are more comprehensive than the current ones. Long reads theoretically confer huge advantages over short reads for transcript-level differential expression, however the low-level of replication and modest read counts obtained from long-read transcriptomic experiments are currently limiting. Until throughput increases and price decreases sufficiently, hybrid approaches that use long reads to define the isoforms expressed in the samples and short reads to get enough counts for well-powered differential expression may be successful; these do not yet exist.

Long-read sequencing technologies have already opened exciting avenues in genomics. Taking on the challenge of obtaining phased, accurate, and complete (including base modifications) genomes and transcriptomes that can be compared will require continued efforts in developing and benchmarking tools.

## Supplementary information

**Additional file 1** Review history.

## References

[1] Pollard MO, Gurdasani D, Mentzer AJ, Porter T, Sandhu MS (2018). Long reads: their purpose and place. Hum Mol Genet.

[2] Burgess DJ (2018). Genomics: next regeneration sequencing for reference genomes. Nat Rev Genet.

[3] Bentley DR, Balasubramanian S, Swerdlow HP, Smith GP, Milton J, Brown CG (2008). Accurate whole human genome sequencing using reversible terminator chemistry. Nature.

[4] Bentley DR (2006). Whole-genome re-sequencing. Curr Opin Genet Dev.

[5] Goodwin S, McPherson JD, McCombie WR (2016). Coming of age: ten years of next-generation sequencing technologies. Nat Rev Genet.

[6] Jeon SA, Park JL, Kim J-H, Kim JH, Kim YS, Kim JC (2019). Comparison of the MGISEQ-2000 and Illumina HiSeq 4000 sequencing platforms for RNA sequencing. Genomics Inform.

[7] Rothberg JM, Hinz W, Rearick TM, Schultz J, Mileski W, Davey M (2011). An integrated semiconductor device enabling non-optical genome sequencing. Nature.

[8] Quail M, Smith ME, Coupland P, Otto TD, Harris SR, Connor TR (2012). A tale of three next generation sequencing platforms: comparison of Ion torrent, Pacific Biosciences and Illumina MiSeq sequencers. BMC Genomics.

[9] Heather JM, Chain B (2016). The sequence of sequencers: the history of sequencing DNA,. Genomics.

[10] Depledge DP, Srinivas KP, Sadaoka T, Bready D, Mori Y, Placantonakis DG (2019). Direct RNA sequencing on nanopore arrays redefines the transcriptional complexity of a viral pathogen. Nat Commun.

[11] Yuan Y, Bayer PE, Batley J, Edwards D (2017). Improvements in genomic technologies: application to crop genomics. Trends in Biotechnol.

[12] Jain M, Olsen HE, Paten B, Akeson M (2016). The Oxford Nanopore MinION: delivery of nanopore sequencing to the genomics community. Genome Biol.

[13] Rang FJ, Kloosterman WP, de Ridder J (2018). From squiggle to basepair: computational approaches for improving nanopore sequencing read accuracy. Genome Biol.

[14] Merker JD, Wenger AM, Sneddon T, Grove M, Zappala Z, Fresard L (2018). Long-read genome sequencing identifies causal structural variation in a Mendelian disease. Genet Med.

[15] Roberts RJ, Carneiro MO, Schatz MC (2013). The advantages of SMRT sequencing. Genome Biol.

[16] Payne A, Holmes N, Rakyan V, Loose M. Whale watching with BulkVis: a graphical viewer for Oxford Nanopore bulk fast5 files. bioRxiv. 2018:312256. 10.1101/312256.

[17] Jain M, Koren S, Miga KH, Quick J, Rand AC, Sasani TA (2018). Nanopore sequencing and assembly of a human genome with ultra-long reads. Nat Biotechnol.

[18] Wenger AM, Peluso P, Rowell WJ, Chang P-C, Hall RJ, Concepcion GT, Ebler J, Fungtammasan A, Kolesnikov A, Olson ND (2019). Accurate circular consensus long-read sequencing improves variant detection and assembly of a human genome. Nat Biotechnol.

[19] McCoy RC, Taylor RW, Blauwkamp TA, Kelley JL, Kertesz M, Pushkarev D, Petrov DA, Fiston-Lavier A-S (2014). Illumina truseq synthetic long-reads empower de novo assembly and resolve complex, highly-repetitive transposable elements. PLoS ONE.

[20] Li R, Hsieh C-L, Young A, Zhang Z, Ren X, Zhao Z (2015). Illumina synthetic long read sequencing allows recovery of missing sequences even in the “finished” *C. elegans* genome. Sci Rep.

[21] Matthews BJ, Dudchenko O, Kingan SB, Koren S, Antoshechkin I, Crawford JE (2018). Improved reference genome of Aedes aegypti informs arbovirus vector control. Nature.

[22] Mortensen Ȯ,., Lydersen LN, Apol KD, Andorsdottir G, Steig B, Gregersen NO (2019). Using dried blood spot samples from a trio for linked-read whole-exome sequencing. Eur J Hum Genet.

[23] Wang O, Chin R, Cheng X, Wu MKY, Mao Q, Tang J (2019). Efficient and unique cobarcoding of second-generation sequencing reads from long DNA molecules enabling cost-effective and accurate sequencing, haplotyping, and de novo assembly. Genome Res.

[24] Senabouth A, Anderson S, Shi Q, Shi L, Jiang F, Zhang W, et al.Comparative performance of the BGI and Illumina sequencing technology for single-cell RNA-sequencing. bioRxiv. 2019:552588. 10.1101/552588. 10.1093/nargab/lqaa034 PMC7671348 33575589

[25] Putnam NH, O’Connell BL, Stites JC, Rice BJ, Blanchette M, Calef R (2016). Chromosome-scale shotgun assembly using an in vitro method for long-range linkage. Genome Res.

[26] Schwartz DC, Li X, Hernandez LI, Ramnarain SP, Huff EJ, Wang YK (1993). Ordered restriction maps of Saccharomyces cerevisiae chromosomes constructed by optical mapping. Science.

[27] Shelton JM, Coleman MC, Herndon N, Lu N, Lam ET, Anantharaman T, Sheth P, Brown SJ (2015). Tools and pipelines for BioNano data: molecule assembly pipeline and FASTA super scaffolding tool. BMC Genomics.

[28] Levy-Sakin M, Ebenstein Y (2013). Beyond sequencing: optical mapping of DNA in the age of nanotechnology and nanoscopy. Curr Opin Biotechnol.

[29] Shi L, Guo Y, Dong C, Huddleston J, Yang H, Han X, Fu A (2016). Long-read sequencing and de novo assembly of a Chinese genome. Nat Commun.

[30] Gordon D, Huddleston J, Chaisson MJP, Hill CM, Kronenberg ZN, Munson KM (2016). Long-read sequence assembly of the gorilla genome. Science.

[31] Mostovoy Y, Levy-Sakin M, Lam J, Lam ET, Hastie AR, Marks P (2016). A hybrid approach for de novo human genome sequence assembly and phasing. Nat Methods.

[32] Weissensteiner MH, Pang AWC, Bunikis I, Höijer I, Vinnere-Petterson O, Suh A (2017). Combination of short-read, long-read, and optical mapping assemblies reveals large-scale tandem repeat arrays with population genetic implications. Genome Res.

[33] Zhu L, Zhong J, Jia X, Liu G, Kang Y, Dong M (2016). Precision methylome characterization of *Mycobacterium tuberculosis* complex (MTBC) using PacBio single-molecule real-time (SMRT) technology. Nucleic Acids Res.

[34] Gigante Scott, Gouil Quentin, Lucattini Alexis, Keniry Andrew, Beck Tamara, Tinning Matthew, Gordon Lavinia, Woodruff Chris, Speed Terence P, Blewitt Marnie E, Ritchie Matthew E (2019). Using long-read sequencing to detect imprinted DNA methylation. Nucleic Acids Research.

[35] Karlsson K, Linnarsson S (2017). Single-cell mRNA isoform diversity in the mouse brain. BMC Genomics.

[36] Byrne A, Beaudin AE, Olsen HE, Jain M, Cole C, Palmer T (2017). Nanopore long-read RNAseq reveals widespread transcriptional variation among the surface receptors of individual B cells. Nat Commun.

[37] Zheng GXY, Lau BT, Schnall-Levin M, Jarosz M, Bell JM, Hindson CM (2016). Haplotyping germline and cancer genomes with high-throughput linked-read sequencing. Nat Biotechnol.

[38] Stapleton JA, Kim J, Hamilton JP, Wu M, Irber LC, Maddamsetti R (2016). Haplotype-phased synthetic long reads from short-read sequencing. PLoS ONE.

[39] Cao H, Wu H, Luo R, Huang S, Sun Y, Tong X (2015). De novo assembly of a haplotype-resolved human genome. Nat Biotechnol.

[40] Kuleshov V, Jiang C, Zhou W, Jahanbani F, Batzoglou S, Snyder M (2016). Synthetic long-read sequencing reveals intraspecies diversity in the human microbiome. Nat Biotechnol.

[41] Nicholls SM, Aubrey W, Edwards A, de Grave K, Huws S, Schietgat L, et al.Computational haplotype recovery and long-read validation identifies novel isoforms of industrially relevant enzymes from natural microbial communities. bioRxiv. 2018. https://doi.org/10.1101/223404. https://www.biorxiv.org/content/early/2018/01/13/223404.full.pdf.

[42] Pacific Biosciences. PacBio RS II workflow. 2015. https://www.pacb.com/wp-content/uploads/2015/09/PacBioWorkflow.pdf. Accessed 20 June 2019.

[43] Pacific Biosciences. Unanimity. 2017. https://github.com/PacificBiosciences/ccs. Accessed 20 June 2019.

[44] Boža V, Brejová B, Vinař T (2017). DeepNano: deep recurrent neural networks for base calling in MinION nanopore reads. PLoS ONE.

[45] Oxford Nanopore Technologies. Oxford Nanopore Technologies GitHub. https://github.com/nanoporetech. Accessed 20 June 2019.

[46] Wick RR, Judd LM, Holt KE (2019). Performance of neural network basecalling tools for Oxford Nanopore sequencing. Genome Biol.

[47] Bowden R, Davies RW, Heger A, Pagnamenta AT, de Cesare M, Oikkonen LE, et al.Sequencing of human genomes with nanopore technology. Nat Commun. 2019; 10(1). 10.1038/s41467-019-09637-5. 10.1038/s41467-019-09637-5 PMC6478738 31015479

[48] Teng H, Cao MD, Hall MB, Duarte T, Wang S, Coin LJM. Chiron: translating nanopore raw signal directly into nucleotide sequence using deep learning. GigaScience. 2018; 7(5). 10.1093/gigascience/giy037. 10.1093/gigascience/giy037 PMC5946831 29648610

[49] de Lannoy C, de Ridder D, Risse J (2017). The long reads ahead: de novo genome assembly using the MinION. F1000Research.

[50] Schmidt MH-W, Vogel A, Denton AK, Istace B, Wormit A, van de Geest H (2017). De novo assembly of a new Solanum pennellii accession using nanopore sequencing. Plant Cell.

[51] Ardui S, Ameur A, Vermeesch JR, Hestand MS (2018). Single molecule real-time (SMRT) sequencing comes of age: applications and utilities for medical diagnostics. Nucleic Acids Res.

[52] Korlach J. Understanding accuracy in SMRT sequencing. Technical report. 2013. www.pacb.com.

[53] Carneiro MO, Russ C, Ross MG, Gabriel SB, Nusbaum C, DePristo MA (2012). Pacific biosciences sequencing technology for genotyping and variation discovery in human data. BMC Genomics.

[54] Weirather JL, de Cesare M, Wang Y, Piazza P, Sebastiano V, Wang X-J (2017). Comprehensive comparison of Pacific Biosciences and Oxford Nanopore Technologies and their applications to transcriptome analysis. F1000Research.

[55] Oxford Nanopore Technologies. Refuelling a sequencing run. 2019. https://community.nanoporetech.com/posts/refuelling-a-sequencing-ru. Accessed 12 Dec 2019.

[56] Oxford Nanopore Technologies. Clive Brown’s keynote at Nanopore Community Meeting 2018. 2018. https://nanoporetech.com/resource-centre/clive-brown-ncm-2018. Accessed 6 June 2019.

[57] Volden R, Palmer T, Byrne A, Cole C, Schmitz RJ, Green RE, Vollmers C (2018). Improving nanopore read accuracy with the R2C2 method enables the sequencing of highly multiplexed full-length single-cell cDNA,. Proc Natl Acad Sci.

[58] Wilson BD, Eisenstein M, Soh HT (2019). High-fidelity nanopore sequencing of ultra-short DNA targets. Anal Chem.

[59] Li C, Chng KR, Boey EJH, Ng AHQ, Wilm A, Nagarajan N (2016). INC-Seq: accurate single molecule reads using nanopore sequencing. GigaScience.

[60] Zhang H, Jain C, Aluru S. A comprehensive evaluation of long read error correction methods. bioRxiv. 2019. https://doi.org/10.1101/519330. https://www.biorxiv.org/content/early/2019/01/13/519330.full.pdf. 10.1186/s12864-020-07227-0 PMC7751105 33349243

[61] Fu S, Wang A, Au KF (2019). A comparative evaluation of hybrid error correction methods for error-prone long reads. Genome Biol.

[62] Lima L, Marchet C, Caboche S, Da Silva C, Istace B, Aury J-M, et al.Comparative assessment of long-read error-correction software applied to rna-sequencing data. bioRxiv. 2019. https://doi.org/10.1101/476622. https://www.biorxiv.org/content/early/2019/03/15/476622.full.pdf. 10.1093/bib/bbz058 31232449

[63] Ruan J, Li H. Fast and accurate long-read assembly with wtdbg2. Nat Methods. 2019. 10.1038/s41592-019-0669-3. 10.1038/s41592-019-0669-3 PMC7004874 31819265

[64] Wang JR, Holt J, McMillan L, Jones CD (2018). FMLRC: hybrid long read error correction using an FM-index. BMC Bioinformatics.

[65] Pacific Biosciences. Genomic consensus. 2018. https://github.com/PacificBiosciences/GenomicConsensus. Accessed 20 June 2019.

[66] Simpson JT, Workman RE, Zuzarte PC, David M, Dursi LJ, Timp W (2017). Detecting DNA cytosine methylation using nanopore sequencing. Nat Methods.

[67] Walker BJ, Abeel T, Shea T, Priest M, Abouelliel A, Sakthikumar S (2014). Pilon: an integrated tool for comprehensive microbial variant detection and genome assembly improvement. PLoS ONE.

[68] Vaser R, Sović I, Nagarajan N, Šikić M (2017). Fast and accurate de novo genome assembly from long uncorrected reads. Genome Res.

[69] Koren S, Walenz BP, Berlin K, Miller JR, Bergman NH, Phillippy AM (2017). Canu: scalable and accurate long-read assembly via adaptive k-mer weighting and repeat separation,. Genome Res.

[70] Jansen HJ, Liem M, Jong-Raadsen SA, Dufour S, Weltzien F-A, Swinkels W (2017). Rapid de novo assembly of the European eel genome from nanopore sequencing reads. Sci Rep.

[71] Miller DE, Staber C, Zeitlinger J, Hawley RS (2018). Highly contiguous genome assemblies of 15 drosophila species generated using nanopore sequencing. G3.

[72] Warren René L, Coombe Lauren, Mohamadi Hamid, Zhang Jessica, Jaquish Barry, Isabel Nathalie, Jones Steven J M, Bousquet Jean, Bohlmann Joerg, Birol Inanç (2019). ntEdit: scalable genome sequence polishing. Bioinformatics.

[73] Mahmoud M, Gobet N, Cruz-Dávalos DI, Mounier N, Dessimoz C, Sedlazeck FJ (2019). Structural variant calling: the long and the short of it. Genome Biol.

[74] Mills RE, Walter K, Stewart C, Handsaker RE, Chen K, Alkan C (2011). Mapping copy number variation by population-scale genome sequencing. Nature.

[75] Sakamoto Yoshitaka, Sereewattanawoot Sarun, Suzuki Ayako (2019). A new era of long-read sequencing for cancer genomics. Journal of Human Genetics.

[76] Mitsuhashi Satomi, Matsumoto Naomichi (2019). Long-read sequencing for rare human genetic diseases. Journal of Human Genetics.

[77] Ho SS, Urban AE, Mills RE. Structural variation in the sequencing era,. Nat Rev Genet. 2019. 10.1038/s41576-019-0180-9. 10.1038/s41576-019-0180-9 PMC7402362 31729472

[78] Audano PA, Sulovari A, Graves-Lindsay TA, Cantsilieris S, Sorensen M, Welch AE (2019). Characterizing the major structural variant alleles of the human genome. Cell.

[79] Chaisson MJP, Huddleston J, Dennis MY, Sudmant PH, Malig M, Hormozdiari F (2015). Resolving the complexity of the human genome using single-molecule sequencing. Nature.

[80] Cretu Stancu M, Van Roosmalen MJ, Renkens I, Nieboer MM, Middelkamp S, De Ligt J, et al.Mapping and phasing of structural variation in patient genomes using nanopore sequencing. Nat Commun. 2017; 8(1). 10.1038/s41467-017-01343-4. 10.1038/s41467-017-01343-4 PMC5673902 29109544

[81] De Coster Wouter, De Rijk Peter, De Roeck Arne, De Pooter Tim, D'Hert Svenn, Strazisar Mojca, Sleegers Kristel, Van Broeckhoven Christine (2019). Structural variants identified by Oxford Nanopore PromethION sequencing of the human genome. Genome Research.

[82] Zook JM, Hansen NF, Olson ND, Chapman LM, Mullikin JC, Xiao C, et al.A robust benchmark for germline structural variant detection. bioRxiv. 2019. https://doi.org/10.1101/664623. https://www.biorxiv.org/content/early/2019/06/09/664623.full.pdf.

[83] Yokoyama Toshiyuki T., Kasahara Masahiro (2019). Visualization tools for human structural variations identified by whole-genome sequencing. Journal of Human Genetics.

[84] Wion D, Casadesús J (2006). N6-methyl-adenine: an epigenetic signal for DNA–protein interactions. Nat Rev Microbiol.

[85] Ito S, Shen L, Dai Q, Wu SC, Collins LB, Swenberg JA (2011). Tet proteins can convert 5-methylcytosine to 5-formylcytosine and 5-carboxylcytosine. Science.

[86] Wossidlo M, Nakamura T, Lepikhov K, Marques CJ, Zakhartchenko V, Boiani M (2011). 5-Hydroxymethylcytosine in the mammalian zygote is linked with epigenetic reprogramming. Nat Commun.

[87] Korlach J, Turner SW (2012). Going beyond five bases in DNA sequencing. Curr Opin Struct Biol.

[88] Shen L, Song C-X, He C, Zhang Y (2014). Mechanism and function of oxidative reversal of dna and rna methylation. Ann Rev Biochem.

[89] Schaefer M, Kapoor U, Jantsch MF (2017). Understanding RNA modifications: the promises and technological bottlenecks of the ‘epitranscriptome’. Open Biol.

[90] Schwartz S, Motorin Y (2017). Next-generation sequencing technologies for detection of modified nucleotides in RNAs. RNA Biol.

[91] Mauer J, Luo X, Blanjoie A, Jiao X, Grozhik AV, Patil DP (2017). Reversible methylation of m^6^A _m_ in the 5^′^ cap controls mRNA stability. Nature.

[92] Patil DP, Chen CK, Pickering BF, Chow A, Jackson C, Guttman M (2016). m6A RNA methylation promotes XIST-mediated transcriptional repression. Nature.

[93] Arango D, Sturgill D, Alhusaini N, Dillman AA, Sweet TJ, Hanson G (2018). Acetylation of cytidine in mRNA promotes translation efficiency,. Cell.

[94] Abcam. Antibodies to RNA modifications. 2019. https://www.abcam.com/epigenetics/antibodies-to-rna-modifications. Accessed 24 May 2019.

[95] Müller CA, Boemo MA, Spingardi P, Kessler BM, Kriaucionis S, Simpson JT, Nieduszynski CA (2019). Capturing the dynamics of genome replication on individual ultra-long nanopore sequence reads. Nat Methods.

[96] Frommer M, McDonald LE, Millar DS, Collis CM, Watt F, Grigg GW (1992). A genomic sequencing protocol that yields a positive display of 5-methylcytosine residues in individual DNA strands,. Proc Natl Acad Sci.

[97] Feederle R, Schepers A (2017). Antibodies specific for nucleic acid modifications. RNA Biol.

[98] Flusberg BA, Webster DR, Lee JH, Travers KJ, Olivares EC, Clark TA (2010). Direct detection of DNA methylation during single-molecule, real-time sequencing. Nat Methods.

[99] Beaulaurier J, Zhang X-S, Zhu S, Sebra R, Rosenbluh C, Deikus G, Shen N, Munera D, Waldor MK, Chess A, Blaser MJ, Schadt EE, Fang G (2015). Single molecule-level detection and long read-based phasing of epigenetic variations in bacterial methylomes. Nat Commun.

[100] Saletore Y, Meyer K, Korlach J, Vilfan ID, Jaffrey S, Mason CE (2012). The birth of the epitranscriptome: deciphering the function of rna modifications. Genome Biol.

[101] Vilfan ID, Tsai Y-C, Clark TA, Wegener J, Dai Q, Yi C (2013). Analysis of RNA base modification and structural rearrangement by single-molecule real-time detection of reverse transcription. J Nanobiotechnol.

[102] Pacific Biosciences. Detecting DNA base modifications using single molecule, real-time sequencing. Technical report. 2015. https://www.pacb.com/wpcontent/uploads/2015/09/WP_Detecting_DNA_Base_Modifications_Using_SMRT_ Sequencing.pdf. Accessed 28 Jan 2020.

[103] Feng Z, Fang G, Korlach J, Clark T, Luong K, Zhang X (2013). Detecting dna modifications from smrt sequencing data by modeling sequence context dependence of polymerase kinetic. PLoS Comput Biol.

[104] Pacific Biosciences. Methylome analysis note. 2017. https://github.com/PacificBiosciences/Bioinformatics-Training/wiki/Methylome-Analysis-Technical-Note. Accessed 12 Dec 2019.

[105] Stoiber MH, Quick J, Egan R, Lee JE, Celniker SE, Neely R, Loman N, Pennacchio L, Brown JB. De novo identification of DNA modifications enabled by genome-guided nanopore signal processing. bioRxiv. 2017:094672. 10.1101/094672.

[106] Liu Q, Georgieva DC, Egli D, Wang K (2019). NanoMod: a computational tool to detect DNA modifications using Nanopore long-read sequencing data. BMC Genomics.

[107] Rand AC, Jain M, Eizenga JM, Musselman-Brown A, Olsen HE, Akeson M (2017). Mapping DNA methylation with high-throughput nanopore sequencing. Nat Methods.

[108] McIntyre ABR, Alexander N, Grigorev K, Bezdan D, Sichtig H, Chiu CY (2019). Single-molecule sequencing detection of N6-methyladenine in microbial reference materials. Nat Commun.

[109] Hennion M, Arbona J-M, Cruaud C, Proux F, Tallec BL, Novikova E, et al.Mapping DNA replication with nanopore sequencing. bioRxiv. 2018. https://doi.org/10.1101/426858. https://www.biorxiv.org/content/early/2018/09/26/426858.full.pdf.

[110] Ni Peng, Huang Neng, Zhang Zhi, Wang De-Peng, Liang Fan, Miao Yu, Xiao Chuan-Le, Luo Feng, Wang Jianxin (2019). DeepSignal: detecting DNA methylation state from Nanopore sequencing reads using deep-learning. Bioinformatics.

[111] Liu Q, Fang L, Yu G, Wang D, Xiao C-l, Wang K (2019). Detection of DNA base modifications by deep recurrent neural network on Oxford Nanopore sequencing data. Nat Commun.

[112] Liu H, Begik O, Lucas MC, Mason CE, Schwartz S, Mattick JS, et al.Accurate detection of m6a RNA modifications in native RNA sequences. bioRxiv. 2019. https://doi.org/10.1101/525741. https://www.biorxiv.org/content/early/2019/01/21/525741.full.pdf. 10.1038/s41467-019-11713-9 PMC6734003 31501426

[113] Dominissini D, Moshitch-Moshkovitz S, Schwartz S, Salmon-Divon M, Ungar L, Osenberg S (2012). Topology of the human and mouse m6a rna methylomes revealed by m6a-seq. Nature.

[114] Meyer KD, Saletore Y, Zumbo P, Elemento O, Mason CE, Jaffrey SR (2012). Comprehensive analysis of mrna methylation reveals enrichment in 3 ^′^ UTRs and near stop codons. Cell.

[115] Mudge JM, Frankish A, Fernandez-Banet J, Alioto T, Derrien T, Howald C, Reymond A, Guigó R, Hubbard T, Harrow J (2011). The origins, evolution, and functional potential of alternative splicing in vertebrates,. Mol Biol Evol.

[116] Frankish A, Mudge JM, Thomas M, Harrow J (2012). The importance of identifying alternative splicing in vertebrate genome annotation,. Database J Biol Databases Curation.

[117] Pan Q, Shai O, Lee LJ, Frey BJ, Blencowe BJ (2008). Deep surveying of alternative splicing complexity in the human transcriptome by high-throughput sequencing. Nat Genet.

[118] Wang ET, Sandberg R, Luo S, Khrebtukova I, Zhang L, Mayr C (2008). Alternative isoform regulation in human tissue transcriptomes. Nature.

[119] Park E, Pan Z, Zhang Z, Lin L, Xing Y (2018). The expanding landscape of alternative splicing variation in human populations. Am J Hum Genet.

[120] Steijger T, Abril JF, Engström PG, Kokocinski F, Akerman M, Alioto T (2013). Assessment of transcript reconstruction methods for RNA-seq. Nat Methods.

[121] Tilgner H, Grubert F, Sharon D, Snyder MP (2014). Defining a personal, allele-specific, and single-molecule long-read transcriptome. Proc Natl Acad Sci.

[122] Au KF, Sebastiano V, Afshar PT, Durruthy JD, Lee L, Williams BA (2013). Characterization of the human ESC transcriptome by hybrid sequencing. Proc Natl Acad Sci.

[123] Clark M, Wrzesinski T, Garcia-Bea A, Kleinman J, Hyde T, Weinberger D, et al.Long-read sequencing reveals the splicing profile of the calcium channel gene CACNA1C in human brain. bioRxiv. 2018:260562. 10.1101/260562.

[124] Sharon D, Tilgner H, Grubert F, Snyder M (2013). A single-molecule long-read survey of the human transcriptome. Nat Biotechnol.

[125] Gupta I, Collier PG, Haase B, Mahfouz A, Joglekar A, Floyd T (2018). Single-cell isoform RNA sequencing characterizes isoforms in thousands of cerebellar cells. Nat Biotechnol.

[126] Tilgner H, Jahanbani F, Blauwkamp T, Moshrefi A, Jaeger E, Chen F (2015). Comprehensive transcriptome analysis using synthetic long-read sequencing reveals molecular co-association of distant splicing events. Nat Biotechnol.

[127] Gonzalez-Garay Manuel L. (2015). Introduction to Isoform Sequencing Using Pacific Biosciences Technology (Iso-Seq). Translational Bioinformatics.

[128] Gordon SP, Tseng E, Salamov A, Zhang J, Meng X, Zhao Z (2015). Widespread polycistronic transcripts in fungi revealed by single-molecule mRNA sequencing. PLoS ONE.

[129] Tseng E. cDNA Cupcake. 2018. https://github.com/Magdoll/cDNA_Cupcake. Accessed 20 June 2019.

[130] Sahlin K, Tomaszkiewicz M, Makova KD, Medvedev P (2018). Deciphering highly similar multigene family transcripts from Iso-Seq data with IsoCon. Nat Commun.

[131] Tardaguila M, de la Fuente L, Marti C, Pereira C, Pardo-Palacios FJ, del Risco H (2018). SQANTI: extensive characterization of long-read transcript sequences for quality control in full-length transcriptome identification and quantification. Genome Res.

[132] Wyman D, Balderrama-Gutierrez G, Reese F, Jiang S, Rahmanian S, Zeng W, et al.A technology-agnostic long-read analysis pipeline for transcriptome discovery and quantification. bioRxiv. 2019. https://doi.org/10.1101/672931. https://www.biorxiv.org/content/early/2019/06/18/672931.full.pdf.

[133] Tang AD, Soulette CM, van Baren MJ, Hart K, Hrabeta-Robinson E, Wu CJ, et al.Full-length transcript characterization of SF3B1 mutation in chronic lymphocytic leukemia reveals downregulation of retained introns. bioRxiv. 2018:410183. 10.1101/410183. 10.1038/s41467-020-15171-6 PMC7080807 32188845

[134] GenomeRIK. Transcriptome annotation by modular algorithm. 2018. https://github.com/GenomeRIK/tama. Accessed 20 June 2019.

[135] Abdel-Ghany SE, Hamilton M, Jacobi JL, Ngam P, Devitt N, Schilkey F (2016). A survey of the sorghum transcriptome using single-molecule long reads,. Nat Commun.

[136] Oxford Nanopore Technologies. Pinfish. 2018. https://github.com/nanoporetech/pinfish. Accessed 20 June 2019.

[137] Kellner S, Burhenne J, Helm M (2010). Detection of RNA modifications. RNA Biol.

[138] Patro R, Duggal G, Love MI, Irizarry RA, Kingsford C (2017). Salmon provides fast and bias-aware quantification of transcript expression. Nat Methods.

[139] Oxford Nanopore Technologies. Wub. 2018. https://github.com/nanoporetech/wub. Accessed 20 June 2019.

[140] Liao Y, Smyth GK, Shi W (2014). featureCounts: an efficient general purpose program for assigning sequence reads to genomic features. Bioinformatics.

[141] Liao Y, Smyth GK, Shi W (2013). The subread aligner: fast, accurate and scalable read mapping by seed-and-vote. Nucleic Acids Res.

[142] Liao Y, Smyth GK, Shi W (2019). The R package Rsubread is easier, faster, cheaper and better for alignment and quantification of RNA sequencing reads. Nucleic Acids Res.

[143] Ritchie ME, Phipson B, Wu D, Hu Y, Law CW, Shi W (2015). limma powers differential expression analyses for RNA-sequencing and microarray studies. Nucleic Acids Res.

[144] Robinson MD, McCarthy DJ, Smyth GK (2010). edgeR: a Bioconductor package for differential expression analysis of digital gene expression data. Bioinformatics.

[145] McCarthy DJ, Chen Y, Smyth GK (2012). Differential expression analysis of multifactor RNA-Seq experiments with respect to biological variation. Nucleic Acids Res.

[146] Love MI, Huber W, Anders S (2014). Moderated estimation of fold change and dispersion for rna-seq data with deseq2. Genome Biol.

[147] Nowicka M, Robinson MD (2016). DRIMSeq: a Dirichlet-multinomial framework for multivariate count outcomes in genomics. F1000Research.

[148] Quick J, Ashton P, Calus S, Chatt C, Gossain S, Hawker J (2015). Rapid draft sequencing and real-time nanopore sequencing in a hospital outbreak of salmonella. Genome Biol.

[149] Berlin K, Koren S, Chin C-S, Drake JP, Landolin JM, Phillippy AM (2015). Assembling large genomes with single-molecule sequencing and locality-sensitive hashing. Nat Biotechnol.

[150] Koren S, Phillippy AM (2015). One chromosome, one contig: complete microbial genomes from long-read sequencing and assembly. Curr Opin Microbiol.

[151] Mahmoud M, Zywicki M, Twardowski T, Karlowski WM (2019). Efficiency of PacBio long read correction by 2nd generation Illumina sequencing. Genomics.

[152] Pennisi E (2017). New technologies boost genome quality. Science.

[153] De Maio N, Shaw LP, Hubbard A, George S, Sanderson ND, Swann J, et al.Comparison of long-read sequencing technologies in the hybrid assembly of complex bacterial genomes. Microb Genom. 2019; 5(9). 10.1099/mgen.0.000294. 10.1099/mgen.0.000294 PMC6807382 31483244

[154] Ellison Christopher E, Cao Weihuan (2019). Nanopore sequencing and Hi-C scaffolding provide insight into the evolutionary dynamics of transposable elements and piRNA production in wild strains of Drosophila melanogaster. Nucleic Acids Research.

[155] Jiao WB, Accinelli GG, Hartwig B, Kiefer C, Baker D, Severing E (2017). Improving and correcting the contiguity of long-read genome assemblies of three plant species using optical mapping and chromosome conformation capture data. Genome Res.

[156] Ma ZS, Li L, Ye C, Peng M, Zhang Y-P (2019). Hybrid assembly of ultra-long nanopore reads augmented with 10x-genomics contigs: demonstrated with a human genome. Genomics.

[157] Jung H, Winefield C, Bombarely A, Prentis P, Waterhouse P (2019). Tools and strategies for long-read sequencing and de novo assembly of plant genomes. Trends Plant Sci.

[158] Miller JR, Zhou P, Mudge J, Gurtowski J, Lee H, Ramaraj T (2017). Hybrid assembly with long and short reads improves discovery of gene family expansions. BMC Genomics.

[159] Beaulaurier John, Schadt Eric E., Fang Gang (2018). Deciphering bacterial epigenomes using modern sequencing technologies. Nature Reviews Genetics.

[160] Wick RR, Judd LM, Gorrie CL, Holt KE (2017). Unicycler: resolving bacterial genome assemblies from short and long sequencing reads. PLoS Comput Biol.

[161] Amarasinghe SL. long_read_tools. 2018. https://github.com/shaniAmare/long_read_tools. Accessed 28 Jan 2020.

[162] Shafin K, Pesout T, Lorig-Roach R, Haukness M, Olsen HE, Bosworth C, et al.Efficient de novo assembly of eleven human genomes using promethion sequencing and a novel nanopore toolkit. bioRxiv. 2019. https://doi.org/10.1101/715722. https://www.biorxiv.org/content/early/2019/07/26/715722.full.pdf.

[163] Chin C-S, Khalak A. Human genome assembly in 100 minutes. bioRxiv. 2019. https://doi.org/10.1101/705616. https://www.biorxiv.org/content/early/2019/07/17/705616.full.pdf.

[164] Kolmogorov M, Yuan J, Lin Y, Pevzner PA (2019). Assembly of long, error-prone reads using repeat graphs. Nat Biotechnol.

[165] Vaser R, Šikić M. Yet another de novo genome assembler. bioRxiv. 2019. https://doi.org/10.1101/656306. https://www.biorxiv.org/content/early/2019/06/08/656306.full.pdf.

[166] Gamaarachchi H. f5c. 2019. https://github.com/hasindu2008/f5c. Accessed 12 Dec 2019.

[167] Watson M, Warr A (2019). Errors in long-read assemblies can critically affect protein prediction. Nat Biotechnol.

[168] Noakes MT, Brinkerhoff H, Laszlo AH, Derrington IM, Langford KW, Mount JW, Bowman JL, Baker KS, Doering KM, Tickman BI, Gundlach JH (2019). Increasing the accuracy of nanopore DNA sequencing using a time-varying cross membrane voltage. Nat Biotechnol.

[169] Hoang NV, Furtado A, Mason PJ, Marquardt A, Kasirajan L, Thirugnanasambandam PP (2017). A survey of the complex transcriptome from the highly polyploid sugarcane genome using full-length isoform sequencing and de novo assembly from short read sequencing. BMC Genomics.

[170] Zhang S-J, Wang C, Yan S, Fu A, Luan X, Li Y (2017). Isoform evolution in primates through independent combination of alternative RNA processing events. Mol Biol Evol.

[171] Soneson C, Yao Y, Bratus-Neuenschwander A, Patrignani A, Robinson MD, Hussain S (2019). A comprehensive examination of Nanopore native RNA sequencing for characterization of complex transcriptomes. Nat Commun.

[172] Sessegolo C, Cruaud C, Da Silva C, Cologne A, Dubarry M, Derrien T, et al.Transcriptome profiling of mouse samples using nanopore sequencing of cDNA and RNA molecules. bioRxiv. 2019. https://doi.org/10.1101/575142. https://www.biorxiv.org/content/early/2019/07/16/575142.full.pdf. 10.1038/s41598-019-51470-9 PMC6797730 31624302

[173] Workman RE, Tang AD, Tang PS, Jain M, Tyson JR, Zuzarte PC, et al.Nanopore native RNA sequencing of a human poly(A) transcriptome. bioRxiv. 2018:459529. 10.1101/459529.

