# Supplementary material for: Opportunities and challenges in long-read sequencing data analysis
Source: Genome Biol. 2020 Feb 7;21:30. doi: 10.1186/s13059-020-1935-5 (PMC7006217; doi:10.1186/s13059-020-1935-5)
Supplement: Supplementary file 1 — Additional file 1 Review history. [file 13059_2020_1935_MOESM1_ESM.docx]

Review History

**First round of review**

**Reviewer 1**

**Comments to author:**

The authors provide a review of PacBio and Oxford Nanopore sequencing platforms (SMRT Sequencing and Nanopore Sequencing, respectively). The authors provide a brief summary of how the platforms work and describe in detail the types of analyses a researcher might be interested in performing once obtaining said data. The authors also present a database that tracks software tools for analyzing long-read sequencing data.

Major Comments

1. In my opinion, in order to be considered a review paper, this manuscript should review SMRT sequencing and Nanopore sequencing along with their subsequent analyses, and then separately, perhaps in 1-2 paragraphs, note the contribution of long-read-tools.org. The current format is a bit confusing - at times it feels like a review, while at other times it feels like an advertisement for their database.

2. Long-read-tools.org is a nice idea. I think it is helpful for new researchers entering a field to have a central database of tools to draw from. As it is, long-read-tools.org has a few interesting features, and is fairly straightforward to navigate. However, I think a bulk of what would make it useful to a researcher is missing. It would be helpful to additionally provide: 1) the underlying algorithms the software use 2) the underlying assumptions for using this software 3) strengths and/or weaknesses of the software 4) overall, how does this software perform. I think it is outside of the scope for the authors to benchmark every single tool themselves, but their database could serve as a central repository for this crowdsourcing kind of information (or minimally showing key results from the publications). Without this information, it is hard for a user to know which of the dozens of tools listed for a specific task they should pick for their application.

3. One of the major uses of long-read sequencing technologies is for structural variant detection. You mention this in the introduction, but do not describe any approaches or software for this application in the body of the manuscript. Please add this.

4. Please be consistent when using company names, instrument names, or sequencing approach names. I notice that you switch back and forth throughout (E.g. comparing PacBio to nanopore sequencing - which is comparing a company to a sequencing approach and should not be done), which could be confusing. I'd recommend clarifying the relationship at the beginning and then sticking with one (ideally, the sequencing approach) throughout.

5. Overall, this article should provide more insights. As it is, this serves as one giant google search for the names of tools. It would be helpful to read a bit more about which tools are better for which circumstances? Or which take longer or more memory? Which are easy to run, etc. Essentially, provide some insights that would help a reader figure out which tools to investigate next for their application, rather than providing them with a list of 300 tools to google.

6. Mostly, this is a very well written article. There are a few places where the authors use technical jargon or specific version names without describing them first that I think would be very confusing and disorienting for a non-expert reader. I've flagged these below - I think this is particularly important for a review article.

Minor Comments

1. [Line 6] - it seems worth mentioning Ion Torrent's platform too as another major player in this space

2. [Lines 5-8] You say that "long-read sequencing technologies generate reads in excess of 10 kilobases". I think this is a bit confusing - are you saying this is the average or the minimum? Certainly the reads are often shorter than 10kb for certain applications. This is specially confusing after providing an "up to" estimate for short reads in lines 5-6. In general, when it comes to giving sizes, I think it is nice to provide an average (or range for what is typical) and then also describe the extremes - just be clear about which you are intending.

3. [Line 36] You say "ONT devices (MinION, GridION, and PromethION)" … please and "and others" since ONT has several other devices. As mentioned above, it would be better to just refer to the sequencing approach rather than the instruments.

4. [Line 37 - 38] Please provide more explanation here (e.g. different bases correspond to different current fluctuations can be identified, etc)

5. [Line 39] should say "the addition of one specific nucleotide"

6. [Line 40] At the end of the sentence "… tiny well". This is a good place to discuss how the approaches give rise to the corresponding strengths and weaknesses (e.g. Nanopore can sequence as long as the input molecule, Both struggle with homopolymers, etc)

7. [Line 40 + Line 43] here you provide the longest read produced by nanopore sequencing and then mention the average SMRT sequencing polymerase length. Similar to above, please stick to one (or both) metrics (e.g. max or average) for both platforms - otherwise it comes across as an unfair comparison.

8. [Line 45] should say "and the raw base error-rate is…"

9. [Line 49] Describe why tools from other approaches are included in the database.

10. [Line 51] If you are going to introduce long-read-tools.org here, please use a section heading to be clear that you are switching from "review" to "introducing something new". As mentioned above, I'd recommend moving all notes about the website to one section rather than sprinkling them in throughout.

11. [Line 81] what does "more flexibility" mean here?

12. [Line 82] What is the reader supposed to surmise from noting the number of tools for each topic? Does the number relate to how advanced the field is? How challenging the topic is? It feels very distracting to me. If you want to include metrics like this, I think it would be better in one central table rather than placed throughout.

13. [Line 83] I think it is worth noting that this data type is a movie - definitely different than other sequencing data types.

14. [Line 86] You need to define the word subread before using it here. Otherwise this is confusing to the reader.

15. [Line 86] You have not explained that the fragments are circularized, so the term "pass of the fragment" doesn't have any context or make sense yet. Please revise.

16. [Line 87] This sentence is also unclear to the introductory user - how can subreads be used to generate a consensus circular sequence? Please revise and add more details.

17. [Line 90] why are the fonts different for fast5 and HDF5 but were not for BAM? Please be consistent.

18. [Lines 94-96] I think you can remove this - listing deprecated tools is not particularly useful to the reader.

19. [Line 97] Can you provide the reader with some context about how Guppy is different from development versions? When should a user choose one verses the other? Without these insights, this manuscript is simply listing tools.

20. [Line 98] Is there a reason you only list Chiron here? While for others you list several tools at a time.

21. [Line 99] This is fair, but can you provide a high level summary that would be useful to the reader? Like, is one more accurate? Or at least what dimensions do they vary on [e.g. accuracy, run time, etc].

22. [Line 116] Can you please provide a citation?

23. [Lines 117-120] This is confusing, can you please clarify?

24. [Line 122] Can you define translocation speed?

25. [Line 123] Is decreasing speed good or bad or neither?

26. [Line 124] Can you provide a citation please?

27. [Line 125] What is "median single pass accuracy" - is it the median across one fragment? Or across a whole run? Also, can you provide a more quantitative descriptor rather than "can reach" - does that mean it is the max? Or do you mean that is a typical value?

28. [Line 126-127] Can you please describe the 1D and 1D-squared protocol, briefly, before you give these numbers? I think that will help the reader put the results into better context. Is the 1D-squared protocol still supported?

29. [Lines 136 & 139] - Similarly, a novice reader will be confused by the terms R9 pores and R10 pores, can you briefly explain the difference? Or minimally, just describe that R10 is the version following R9 (I'm not sure that that is intuitive for an unfamiliar reader).

30. [Lines 137] - what do you mean by "dimensions on the pore"

31. [Line 140] - you suddenly jump into discussing "certain k-mers" but have not previously described how k-mer sequences are used for current intensity detection and how it relates to base calling. Please clarify here.

32. [Line 144] When you say ONT "regularly" release chemistry and software updates, it makes it sound like PacBio doesn't (in comparison). Perhaps you can put a frequency value to these? Or if you don't intend to make that comparison, simply say that they are both maintaining and upgrading their chemistry and software.

33. [Line 171] This would be a good place to add a sentence to provide some context about the practicality of sequencing both long and short reads for the same sample (cost, time, input sample, etc… essentially, is this a commonly used protocol or more theoretical? Are there certain cases that lend themselves to this approach more than others?)

34. [Line 172] It is unclear what you mean by polishing. E.g. how do these tools improve consensus accuracy? How to they take into account base modifications?

35. [Line 178] consider adding "and others" after Pilon or Racon.

36. [Line 182] It isn't intuitive why more iterations would decrease the quality of an assembly- can you describe why this is? How many rounds should one use (and how do you know)?

37. [Line 228] Why is the coverage amount required so different? Can you provide some intuition here.

38. [Line 232] Any software to mention here?

39. [Table 1] This seems somewhat random as the first table to include, since you don't include a table for the other topics discussed (e.g. base calling, polishing, etc)

40. [Line 319] Change "sensitivity and accuracy" to "sensitivity and positive predictive value" or simply "accuracy"

41. [Line 357] Most of what is contained in this section is Discussion, not Conclusion.

42. [Line 376] "rate of addition" is a bit odd here as you haven't cited the rate of addition. I think this is a bit too casual / colloquial here - consider simple removing this part of the sentence or saying that your group plans to add to and maintain the database

43. [Line 390] Please provide a citation for Canu

**Reviewer 2**

**Comments to author:**

Dear Authors,

I quite like the manuscript, and the idea behind it. A comprehensive, curated long read tool collection source is useful for the community. I do not have any major comments. My main comments are below:

1) I felt that the manuscript could benefit from a discussion of some platform combination (Illumina + PacBio, Nanopore + Illumina, Nanopore + Illumina + PacBio, etc.).

2) What about the other long read platforms, like HiC, 10X, and Bionano, etc.? I think those deserve some discussion and how well do they work in synergy with single-molecule sequencing.

3) You briefly talked about price, but I think it is worth talking about cost and scale since we are moving towards high-throughput genomes and analyses.

4) There is also a need to talk about timelines for data generation and compute analysis time. For example, nanopore sequencing is very fast in turn around time compared to other platforms. Similar speed comparisons could be drawn for the software, where possible.

I send my best wishes to the authors.

Thank you.

**Authors Response**

**Point-by-point responses to the reviewers’ comments:**

Reviewer reports:

Reviewer #1: The authors provide a review of PacBio and Oxford Nanopore sequencing platforms (SMRT Sequencing and Nanopore Sequencing, respectively). The authors provide a brief summary of how the platforms work and describe in detail the types of analyses a researcher might be interested in performing once obtaining said data. The authors also present a database that tracks software tools for analyzing long-read sequencing data.

Major Comments

1. In my opinion, in order to be considered a review paper, this manuscript should review SMRT sequencing and Nanopore sequencing along with their subsequent analyses, and then separately, perhaps in 1-2 paragraphs, note the contribution of long-read-tools.org. The current format is a bit confusing - at times it feels like a review, while at other times it feels like an advertisement for their database.

*We have now separated the core of the review from the presentation of long-read-tools.org (please see line 445).*

2. Long-read-tools.org is a nice idea. I think it is helpful for new researchers entering a field to have a central database of tools to draw from. As it is, long-read-tools.org has a few interesting features, and is fairly straightforward to navigate. However, I think a bulk of what would make it useful to a researcher is missing. It would be helpful to additionally provide: 1) the underlying algorithms the software use 2) the underlying assumptions for using this software 3) strengths and/or weaknesses of the software 4) overall, how does this software perform. I think it is outside of the scope for the authors to benchmark every single tool themselves, but their database could serve as a central repository for this crowdsourcing kind of information (or minimally showing key results from the publications). Without this information, it is hard for a user to know which of the dozens of tools listed for a specific task they should pick for their application.

*Thank you for this useful feedback. We have added these fields to the database, accessible with the details of the tool, and populated them for the tools that we mention in the review. We expect that more information can be added in time as benchmark papers are published and new tools are submitted.*

3. One of the major uses of long-read sequencing technologies is for structural variant detection. You mention this in the introduction, but do not describe any approaches or software for this application in the body of the manuscript. Please add this.

*We agree structural variation is amongst the most natural applications of long-read sequencing. We have now added a section about structural variant detection, but we keep in short in light of the very recent publication of reviews entirely dedicated to this topic, covering algorithms and long-read sequencing: Ho et al. Nature Reviews Genetics 2019, Mahmoud et al. Genome Biology 2019 (please see line 221).*

4. Please be consistent when using company names, instrument names, or sequencing approach names. I notice that you switch back and forth throughout (E.g. comparing PacBio to nanopore sequencing - which is comparing a company to a sequencing approach and should not be done), which could be confusing. I'd recommend clarifying the relationship at the beginning and then sticking with one (ideally, the sequencing approach) throughout.

*We apologise for the inconsistency, and now adopt the sequencing-based nomenclature (SMRT and nanopore) throughout (please see line 23 for definition).*

5. Overall, this article should provide more insights. As it is, this serves as one giant google search for the names of tools. It would be helpful to read a bit more about which tools are better for which circumstances? Or which take longer or more memory? Which are easy to run, etc. Essentially, provide some insights that would help a reader figure out which tools to investigate next for their application, rather than providing them with a list of 300 tools to google.

*We have expanded the discussions around the tools that are mentioned, and we draw increased attention to the workflow figure that highlights commonly used tools, however a comprehensive benchmark of performance is outside the scope of the review. With the explanation of the principles behind major applications, and the expanded fields in the database (reviewer comment #2), we believe the review cannot be reduced to a mere list of tools. We would also like to note that a simple list would still be useful to the community, as the impact of scrna-tools, the model long-read-tools is based on, attests: its publication has already garnered 51 citations in a little over a year, and its database receives 1,000 visitors a month.*

6. Mostly, this is a very well written article. There are a few places where the authors use technical jargon or specific version names without describing them first that I think would be very confusing and disorienting for a non-expert reader. I've flagged these below - I think this is particularly important for a review article.

*Thank you for the close reading of the manuscripts and the suggestions for improvement.*

Minor Comments

1. [Line 6] - it seems worth mentioning Ion Torrent's platform too as another major player in this space

*We have added a mention of Ion Torrent (please see line 7).*

2. [Lines 5-8] You say that "long-read sequencing technologies generate reads in excess of 10 kilobases". I think this is a bit confusing - are you saying this is the average or the minimum? Certainly the reads are often shorter than 10kb for certain applications. This is specially confusing after providing an "up to" estimate for short reads in lines 5-6. In general, when it comes to giving sizes, I think it is nice to provide an average (or range for what is typical) and then also describe the extremes - just be clear about which you are intending.

*Since this comparison occurs in the introduction before we introduce the long-read sequencing technologies, we clarify this statement (“long-read sequencing technologies routinely generate reads in excess of 10 kilobases”; please see line 8) and provide ranges in the following section, “state of long-read sequencing and data analysis”: “The library insert sizes amenable to SMRT sequencing range from 250 bp to 50 kbp. Nanopore sequencing provides the longest read lengths, from 500 bp to the current record of 2.3 Mb, with 10–30 kb genomic libraries being common” (please see line 48).*

3. [Line 36] You say "ONT devices (MinION, GridION, and PromethION)" … please and "and others" since ONT has several other devices. As mentioned above, it would be better to just refer to the sequencing approach rather than the instruments.

*The three sequencers listed are the only ones ONT have commercially released, but the statement was ambiguous as ONT also make other “devices”, e.g. data acquisition units or library preparation systems. We have changed this statement as suggested, by sequencing approach: “Nanopore sequencers (MinION, GridION, and PromethION) can identify [...]. SMRT sequencers (RS II, Sequel and Sequel II) detect [...] ” (please see line 30).*

4. [Line 37 - 38] Please provide more explanation here (e.g. different bases correspond to different current fluctuations can be identified, etc)

*We have added the sentence: “Different nucleotides confer different resistances to the stretch of nucleic acid within the pore, therefore the sequence of bases can be inferred from the specific patterns of current variation” (please see line 41).*

5. [Line 39] should say "the addition of one specific nucleotide"

*We have changed the sentence accordingly (please see line 45).*

6. [Line 40] At the end of the sentence "… tiny well". This is a good place to discuss how the approaches give rise to the corresponding strengths and weaknesses (e.g. Nanopore can sequence as long as the input molecule, Both struggle with homopolymers, etc)

*We have expanded the following paragraph on the technical characteristics of both technologies (read lengths, accuracy) (please see line 47). Homopolymers are discussed in the Error Correction section (please see line 121).*

7. [Line 40 + Line 43] here you provide the longest read produced by nanopore sequencing and then mention the average SMRT sequencing polymerase length. Similar to above, please stick to one (or both) metrics (e.g. max or average) for both platforms - otherwise it comes across as an unfair comparison.

*We have rewritten this section to include further detail and both metrics for both platforms (please see line 49).*

8. [Line 45] should say "and the raw base error-rate is…"

*We have changed the sentence accordingly (please see line 56).*

9. [Line 49] Describe why tools from other approaches are included in the database.

*As we now introduce the database at the end of the review, this section was moved there as well. We explain the inclusion of these tools as a way to make the database more comprehensive and because of their synergistic relationships with true long-read sequencing (Hi-C + nanopore for assembly, etc) (please see line 445).*

10. [Line 51] If you are going to introduce long-read-tools.org here, please use a section heading to be clear that you are switching from "review" to "introducing something new". As mentioned above, I'd recommend moving all notes about the website to one section rather than sprinkling them in throughout.

*As suggested all mentions of the database have been separated from the review and placed at the end (please see line 445).*

11. [Line 81] what does "more flexibility" mean here?

*We intended to convey that there were more options for basecalling nanopore data than SMRT data: production and experimental basecallers from ONT, third party basecallers, canonical or expanded basecallers (e.g. to include modifications), basecallers trained on custom data, etc.*

*We have clarified this by contrasting the number of basecalling tools dedicated to each technology, and give additional details in the following paragraphs (please see line 85).*

12. [Line 82] What is the reader supposed to surmise from noting the number of tools for each topic? Does the number relate to how advanced the field is? How challenging the topic is? It feels very distracting to me. If you want to include metrics like this, I think it would be better in one central table rather than placed throughout.

*We have removed unnecessary mentions of tool numbers, as these are visible in Fig 1. We now only mention numbers when required to contrast nanopore/SMRT, highlight the complexity of the field, etc.*

13. [Line 83] I think it is worth noting that this data type is a movie - definitely different than other sequencing data types.

*We have included this clarification (please see line 88).*

14. [Line 86] You need to define the word subread before using it here. Otherwise this is confusing to the reader.

*We have included a clarification of subreads (please see line 93).*

15. [Line 86] You have not explained that the fragments are circularized, so the term "pass of the fragment" doesn't have any context or make sense yet. Please revise.

*We have edited relevant the paragraph to make this more clear (please see line 88)*

16. [Line 87] This sentence is also unclear to the introductory user - how can subreads be used to generate a consensus circular sequence? Please revise and add more details.

*We have rewritten this paragraph to improve clarity (please see line 88).*

*We note that the consensus is obtained from aligning the subreads together, but the details of how the ccs algorithm works are not public.*

17. [Line 90] why are the fonts different for fast5 and HDF5 but were not for BAM? Please be consistent.

*All file formats are now in typewriter font.*

18. [Lines 94-96] I think you can remove this - listing deprecated tools is not particularly useful to the reader.

*We have changed the sentence accordingly.*

19. [Line 97] Can you provide the reader with some context about how Guppy is different from development versions? When should a user choose one verses the other? Without these insights, this manuscript is simply listing tools.

*We have expanded on the characteristics of various basecallers (please see line 106).*

20. [Line 98] Is there a reason you only list Chiron here? While for others you list several tools at a time.

*We single out Chiron because outside of the proprietary basecallers it is the most prominent (tested externally, and not deprecated). We have change “for example Chiron” to “most prominently Chiron” to reflect this (please see line 111).*

21. [Line 99] This is fair, but can you provide a high level summary that would be useful to the reader? Like, is one more accurate? Or at least what dimensions do they vary on [e.g. accuracy, run time, etc].

*We now mention that Guppy was found to have a good overall performance, with good accuracy and fast execution. We also note that nanopore basecalling remains an area of active research (please see line 110).*

22. [Line 116] Can you please provide a citation?

*Citation added. Thank you for spotting it.*

23. [Lines 117-120] This is confusing, can you please clarify?

*We have added one sentence to clarify the consequences of random vs non-random errors in raw reads: “If the errors were non-random, increasing the sequencing depth would not be sufficient to remove them. However the randomness of errors in subreads [...]” (please see line 131).*

24. [Line 122] Can you define translocation speed?

*We have added a definition: “the rate of ratcheting base by base” (please see line 139).*

25. [Line 123] Is decreasing speed good or bad or neither?

*Definitely bad. We now spell this out (please see line 141) .*

26. [Line 124] Can you provide a citation please?

*CItation added, thank you for spotting it.*

27. [Line 125] What is "median single pass accuracy" - is it the median across one fragment? Or across a whole run? Also, can you provide a more quantitative descriptor rather than "can reach" - does that mean it is the max? Or do you mean that is a typical value?

*We clarify that it is the median across a run, and use “can reach” to highlight that these are manufacturer’s numbers, under optimal conditions. We then contrast the number with the same statistic “in the field” (please see line 147).*

28. [Line 126-127] Can you please describe the 1D and 1D-squared protocol, briefly, before you give these numbers? I think that will help the reader put the results into better context. Is the 1D-squared protocol still supported?

*We now introduce the two sequencing strategies before providing accuracy numbers: “In the most common, 1D sequencing protocol, each strand of the dsDNA fragment is read independently, and this single pass accuracy is the final accuracy for the fragment. By contrast, the 1D^2 protocol is designed to sequence the complementary strand in immediate succession of up to 75% of fragments, which allows the calculation of a more accurate consensus sequence for the library insert”. 2D sequencing has been discontinued but 1D^2 is supported (please see line 142).*

29. [Lines 136 & 139] - Similarly, a novice reader will be confused by the terms R9 pores and R10 pores, can you briefly explain the difference? Or minimally, just describe that R10 is the version following R9 (I'm not sure that that is intuitive for an unfamiliar reader).

*We now make clear that R9 pores are the current standard, and that the newer (R10) pores are designed to address the homopolymer problem (please see line 159).*

30. [Lines 137] - what do you mean by "dimensions on the pore"

*This was ambiguous and the behaviour at homopolymers actually depends on more than the length of the channel (where the constriction points are is important too), so we have removed this statement. Instead, we explain why homopolymers are difficult to measure: “Measured current is a function of the particular k-mer residing in the pore, and because translocation of homopolymers does not change the sequence of nucleotides within the pore, it results in a constant signal that makes determining homopolymer length difficult.” (please see line 159).*

31. [Line 140] - you suddenly jump into discussing "certain k-mers" but have not previously described how k-mer sequences are used for current intensity detection and how it relates to base calling. Please clarify here.

*This is now detailed as above (please see line 159).*

32. [Line 144] When you say ONT "regularly" release chemistry and software updates, it makes it sound like PacBio doesn't (in comparison). Perhaps you can put a frequency value to these? Or if you don't intend to make that comparison, simply say that they are both maintaining and upgrading their chemistry and software.

*Thank you for pointing this out, we have added frequencies for both companies:*

*“ONT regularly release chemistry and software updates that improve read quality: 4 pore versions were introduced in the last three years (R9.4, R9.4.1, R9.5.1, R10.0), and in 2019 only there were 12 Guppy releases. PacBio similarly updates hardware, chemistry and software: the last three years have seen the release of 1 instrument (Sequel II), 4 chemistries (Sequel v2 and v3, Sequel II v1 and v2) and 4 versions of the SMRT-LINK analysis suite.” (please see line 168).*

33. [Line 171] This would be a good place to add a sentence to provide some context about the practicality of sequencing both long and short reads for the same sample (cost, time, input sample, etc… essentially, is this a commonly used protocol or more theoretical? Are there certain cases that lend themselves to this approach more than others?)

*As hybrid (long and short) approaches are relevant to multiple applications, we have added these points to the discussion (please see line 505).*

34. [Line 172] It is unclear what you mean by polishing. E.g. how do these tools improve consensus accuracy? How to they take into account base modifications?

*We have made the distinction between error correction (raw reads) and polishing (contigs) clearer (please see line 198).*

35. [Line 178] consider adding "and others" after Pilon or Racon.

*This suggested change was made (please see line 204).*

36. [Line 182] It isn't intuitive why more iterations would decrease the quality of an assembly- can you describe why this is? How many rounds should one use (and how do you know)?

*It is indeed unintuitive and not well understood. We now indicate that the BUSCO score may be used to determine the accuracy of an assembly, and the appropriate number of polishing iterations (please see line 209).*

37. [Line 228] Why is the coverage amount required so different? Can you provide some intuition here.

*We now explain that the increased coverage requirement stems from the subtler impacts 5mC and hmC have on polymerase kinetics, compared to 4mC and 6mA (please see line 286).*

38. [Line 232] Any software to mention here?

*We now mention a few options: the SMRT portal, R-kinetics, kineticsTools, basemods and SMALR (please see line 290).*

39. [Table 1] This seems somewhat random as the first table to include, since you don't include a table for the other topics discussed (e.g. base calling, polishing, etc)

*We have chosen to include a table in the case of long-read epigenetics as the principles are radically new compared to short-read sequencing (unlike polishing for instance), there are very recent developments and the tools vary in the modifications they can detect, making a quick look up potentially useful.*

40. [Line 319] Change "sensitivity and accuracy" to "sensitivity and positive predictive value" or simply "accuracy"

*Replaced with accuracy (please see line 380).*

41. [Line 357] Most of what is contained in this section is Discussion, not Conclusion.

*The section has been renamed as Discussion (please see line 468).*

42. [Line 376] "rate of addition" is a bit odd here as you haven't cited the rate of addition. I think this is a bit too casual / colloquial here - consider simple removing this part of the sentence or saying that your group plans to add to and maintain the database

*We have removed this part of the sentence.*

43. [Line 390] Please provide a citation for Canu

*Citation added. Thank you for spotting this.*

-----------------------------------------

Reviewer #2: Dear Authors,

I quite like the manuscript, and the idea behind it. A comprehensive, curated long read tool collection source is useful for the community. I do not have any major comments. My main comments are below:

1) I felt that the manuscript could benefit from a discussion of some platform combination (Illumina + PacBio, Nanopore + Illumina, Nanopore + Illumina + PacBio, etc.).

*We have added a section dedicated to hybrid approaches (please see line 418).*

2) What about the other long read platforms, like HiC, 10X, and Bionano, etc.? I think those deserve some discussion and how well do they work in synergy with single-molecule sequencing.

*These technologies are now discussed in the “hybrid approaches” section, and we point out that these are represented in the long-read-tools database (please see line 418).*

3) You briefly talked about price, but I think it is worth talking about cost and scale since we are moving towards high-throughput genomes and analyses.

*Thank you for the suggestion, we have expanded on this issue in the discussion (please see line 468 onwards).*

4) There is also a need to talk about timelines for data generation and compute analysis time. For example, nanopore sequencing is very fast in turn around time compared to other platforms. Similar speed comparisons could be drawn for the software, where possible.

*We have added mentions of software speed in the relevant sections, and discuss turnaround time along with the cost and scalability.*

**Second round of review**

**Reviewer 1**

The authors have addressed my concerns - thank you.
